# High-Resolution Mapping of H1 Linker Histone Variants in Embryonic Stem Cells

**DOI:** 10.1371/journal.pgen.1003417

**Published:** 2013-04-25

**Authors:** Kaixiang Cao, Nathalie Lailler, Yunzhe Zhang, Ashwath Kumar, Karan Uppal, Zheng Liu, Eva K. Lee, Hongwei Wu, Magdalena Medrzycki, Chenyi Pan, Po-Yi Ho, Guy P. Cooper, Xiao Dong, Christoph Bock, Eric E. Bouhassira, Yuhong Fan

**Affiliations:** 1 School of Biology, Georgia Institute of Technology, Atlanta, Georgia, United States of America; 2 Parker H. Petit Institute for Bioengineering and Bioscience, Georgia Institute of Technology, Atlanta, Georgia, United States of America; 3 Department of Medicine, Albert Einstein College of Medicine, Bronx, New York, United States of America; 4 School of Industrial and Systems Engineering, Georgia Institute of Technology, Atlanta, Georgia, United States of America; 5 School of Electrical and Computer Engineering, Georgia Institute of Technology, Atlanta, Georgia, United States of America; 6 CeMM Research Center for Molecular Medicine of the Austrian Academy of Sciences, Vienna, Austria; 7 Department of Laboratory Medicine, Medical University of Vienna, Vienna, Austria; 8 Max Planck Institute for Informatics, Saarbrücken, Germany; Medical Research Council Human Genetics Unit, United Kingdom

## Abstract

H1 linker histones facilitate higher-order chromatin folding and are essential for mammalian development. To achieve high-resolution mapping of H1 variants H1d and H1c in embryonic stem cells (ESCs), we have established a knock-in system and shown that the N-terminally tagged H1 proteins are functionally interchangeable to their endogenous counterparts *in vivo*. H1d and H1c are depleted from GC- and gene-rich regions and active promoters, inversely correlated with H3K4me3, but positively correlated with H3K9me3 and associated with characteristic sequence features. Surprisingly, both H1d and H1c are significantly enriched at major satellites, which display increased nucleosome spacing compared with bulk chromatin. While also depleted at active promoters and enriched at major satellites, overexpressed H1^0^ displays differential binding patterns in specific repetitive sequences compared with H1d and H1c. Depletion of H1c, H1d, and H1e causes pericentric chromocenter clustering and de-repression of major satellites. These results integrate the localization of an understudied type of chromatin proteins, namely the H1 variants, into the epigenome map of mouse ESCs, and we identify significant changes at pericentric heterochromatin upon depletion of this epigenetic mark.

## Author Summary

Embryonic stem cells (ESCs) possess unique chromatin and epigenetic signatures, which are important in defining the identity and genome plasticity of pluripotent stem cells. Although ESC epigenomes have been extensively characterized, the genome localization of histone H1 variants, the chromatin structural proteins facilitating higher-order chromatin folding, remains elusive. Linker histone H1 is essential for mammalian development and regulates the expression of specific genes in ESCs. Here, by using a knock-in system coupled with ChIP–seq, we first achieve the high resolution mapping of two H1 variants on a genome-wide scale in mouse ESCs. Our study reveals the correlations of this underexplored histone family with other epigenetic marks and genome attributes. Surprisingly, we identify a dramatic enrichment of H1d and H1c at major satellite sequences. H1^0^, mapped using an overexpressing ESC line, shows similar features at active promoters but differential binding at repetitive sequences compared with H1d and H1c. Furthermore, using mutant ESCs that are deficient for multiple H1 variants, we demonstrate the role of H1 in chromocenter clustering and transcriptional repression of major satellites. Thus, these results connect this important repressive mark with the well understood ESC epigenome and identify novel functions of H1 in mammalian genome organization.

## Introduction

In all eukaryotes, nuclear DNA is packaged into chromatin by its association with histones [1]. The nucleosome, the building block of chromatin, consists of an octamer of four core histones (H2A, H2B, H3 and H4) wrapped by 147 bp of DNA [2]. Linker histone H1 binds to DNA entering and exiting nucleosome core particles as well as the linker DNA between nucleosomes, facilitating folding of chromatin into higher order structure [1], [3]–[5].

The H1 histone family is the most divergent group among the highly conserved histone proteins. To date, 11 different H1 variants have been characterized in mammals, including somatic H1 variants (H1a to H1e), the replacement H1 (H1^0^), germ cell specific H1s (H1t, H1T2, HILS1 and H1oo), as well as the recently characterized variant H1x [6]. Deletion of three major somatic H1 variants (H1c, H1d and H1e) together leads to a 50% reduction of the total H1 level and embryonic lethality at midgestation, demonstrating that H1 level is critical for mammalian development [7]. H1 variants are conserved from mouse to human, and differ in their biochemical properties and expression patterns during development and malignant transformation [8]–[11]. Although none of the H1 variants tested is essential for mouse development [12]–[15], they have been shown to regulate specific gene expression in various cell types [6], [16]–[18]. However, the mechanisms by which H1 variants modulate chromatin structure and gene expression remain under-explored. Mapping of the precise genomic localizations of different H1 variants *in vivo* is likely to provide significant insights, but has been challenging due to the lack of high quality antibodies that could accurately distinguish different H1 variants.

Pluripotent embryonic stem cells (ESCs) can differentiate into cells of all three germ layers, offering great potential in regenerative medicine. The epigenome is suggested to play a critical role in stem cell fate determination, and genome-wide mapping studies have revealed that ESCs have characteristic epigenetic landscapes that differ from differentiated cells [19], [20]. However, despite significant efforts to characterize the chromatin features of human and mouse ESCs, both by individual labs [19], [21]–[23] and by large consortia (ENCODE [24], Roadmap Epigenomics [25]), the landscapes of linker histone H1 variants have not been described on a genome-wide scale.

In this study, we have achieved high resolution mapping of H1d, H1c and H1^0^ in ESCs by chromatin immunoprecipitation followed by massive parallel sequencing (ChIP-seq). H1d and H1c are among the most abundant linker histones in mouse ESCs, accounting respectively for 32.6% and 16.4% of total H1, whereas the differentiation associated H1, H1^0^, accounts for 2% of H1 in undifferentiated ESCs [26], [27]. These three variants differ significantly in terms of their residence time on chromatin and their ability to promote chromatin condensation *in vitro* [28], [29]. They also display different expression patterns during mammalian development and in exponentially growing cells *vs.* quiescent cells [8], [10], [30]. Here, we have generated FLAG-tagged H1d knock-in ESCs, Myc-tagged H1c knock-in ESCs, as well as FLAG-tagged H1^0^ overexpressing ESCs, designated as respective H1d^FLAG^, H1c^Myc^, and fH1^0^ cells. We demonstrate that tagged H1 variants maintain the biochemical properties of the endogenous H1s *in vivo* and that FLAG-H1d can substitute for H1d during mouse development. High resolution mapping reveals that H1d and H1c occupancies are highly correlated, both enriched at AT-rich regions, but also possess different binding specificity. Both H1d and H1c largely co-localize with H3K9me3, but show an inverse correlation with GC% or H3K4me3. Importantly, we discover that H1d and H1c are highly enriched at major satellite elements, which display a longer nucleosome repeat length than bulk chromatin in ESCs. Finally, we show that H1 depletion leads to chromocenter clustering and increased expression of major satellites independent of multiple epigenetic marks at these regions.

## Results

### Generation of tagged H1 knock-in ESCs

**Figure 1:**
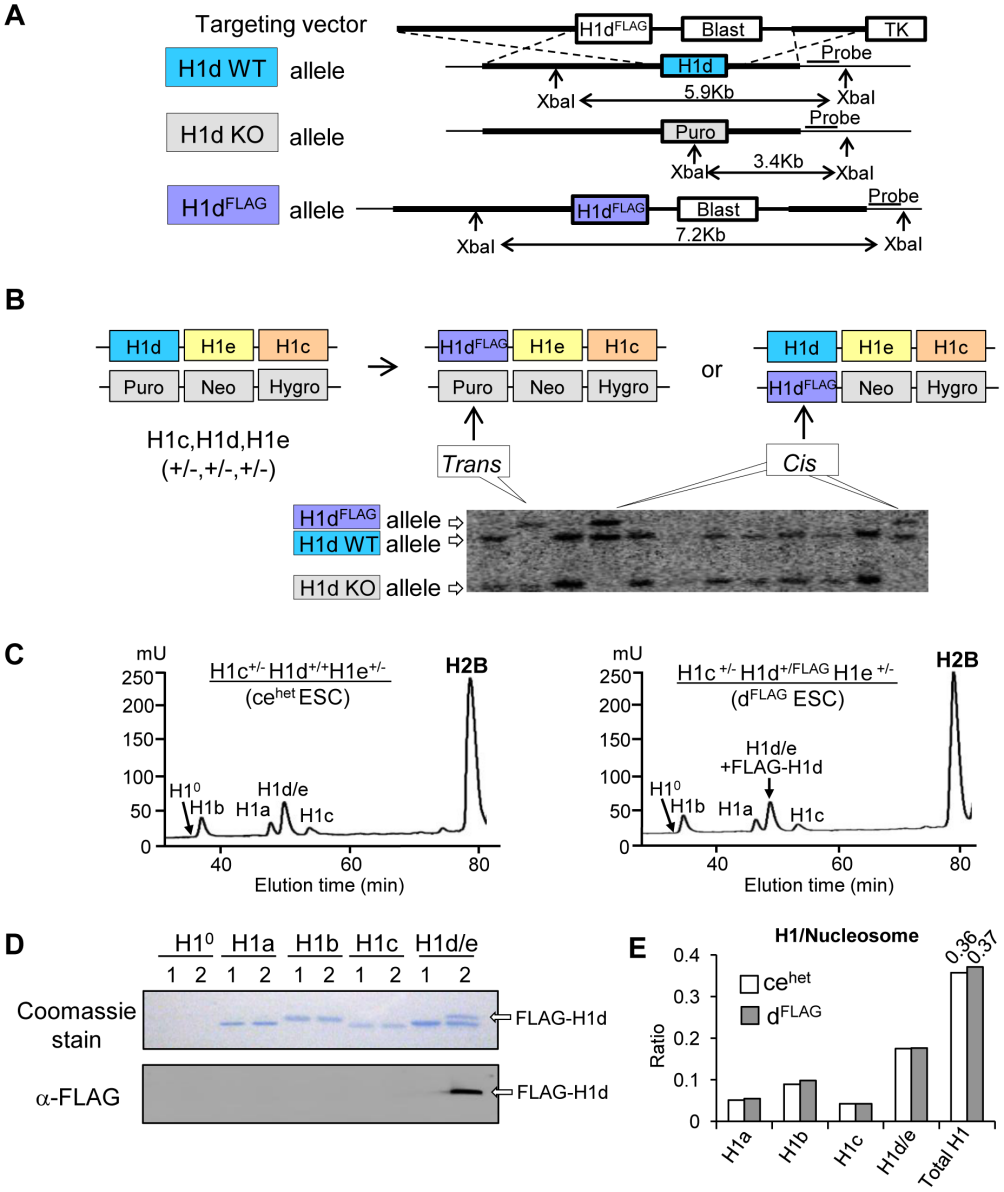
Generation of H1d^FLAG^ knock-in ESCs. (A) Schematic representation of the H1d^FLAG^ targeting construct and the knock-in strategy for insertion of the FLAG tag at N-terminus of the endogenous H1d gene. (B) Identification of ESC clones containing the modified FLAG-H1d allele. DNA isolated from Blasticidin resistant ESC clones were analyzed by Southern blotting. *Cis vs. trans* configurations of the homologous recombination events are schematically illustrated in the diagram above the Southern blotting image. (C) Reverse phase HPLC profiles of histone extracts from ce^het^ (left panel) and *cis*-targeted H1d^FLAG^ ESCs (right panel). mU, milliunits of absorbency at 214 nm. (D) Coomassie staining and Western blotting analysis of individual H1 fractions eluted from HPLC of histone extracts of ce^het^ (1) and H1d^FLAG^ (2) ESCs. (E) Calculated ratio of each H1 variant (and total H1) to nucleosome of ce^het^ and H1d^FLAG^ ESCs.

Efforts to generate high resolution genome-wide maps of H1 variants were hampered by the lack of H1 variant specific antibodies of sufficient quality for ChIP-seq. Here, we established knock-in mouse ESC lines in which H1d or H1c variant was N-terminally tagged with an epitope (FLAG or Myc) for which highly specific antibodies exist. An H1d^FLAG^ cell line was created by inserting the FLAG tag coding sequence at the endogenous H1d locus through homologous recombination (Figure 1A). H1c/H1e double knockout mice develop normally, yet H1c/H1d/H1e triple knockout (H1 TKO) mice are embryonic lethal [7]. Thus, ESCs with H1d^FLAG^ allele in H1c^+/−^H1e^+/−^ background could be used to produce H1c^−/−^H1d^FLAG/FLAG^H1e^−/−^ mice to determine whether FLAG-tagged H1d (FLAG-H1d) functions equivalently to endogenous H1d by assessing if the tagged H1d can rescue the embryonic lethality of H1 TKO mutants. Toward this end, we generated both H1c^+/−^H1d^+/FLAG^H1e^+/−^ (“H1d^FLAG^”) and H1c^+/−^H1d^FLAG/−^H1e^+/−^ (“H1d-*trans*”) ESC lines by transfection of the FLAG-H1d targeting vector (Figure 1A) into the *cis* triply targeted H1c^+/−^H1d^+/−^H1e^+/−^ ESCs established previously [7]. ESC clones with either *cis* or *trans* configuration of the H1d^FLAG^ allele with the H1c and H1e KO allele were identified and verified by Southern blotting (Figure 1B). As expected, FLAG-H1d was located in the nuclei of the H1d^FLAG^ cells (data not shown). Analysis of histone extracts of chromatin prepared from *cis*-targeted H1d^FLAG^ cells by HPLC and immunoblotting indicated that FLAG-H1d was associated with chromatin and eluted in the same fraction as the endogenous H1d, suggesting that FLAG-H1d has the same hydrophobicity as the endogenous H1d (Figure 1C and 1D). The ratio of somatic H1 variants, H1 a–e, to nucleosome (H1/nuc) of H1d^FLAG^ cells was nearly identical to that of H1c^+/−^H1d^+/+^H1e^+/−^ (ce^het^) cells, indicating a similar expression level of FLAG-H1d as the endogenous H1d (Figure 1E). As expected, the protein level of differentiation associated H1^0^ variant was minimal in undifferentiated ESCs. We injected *cis*-targeted H1d^FLAG^ cells into mouse blastocysts and produced chimeric mice which gave germline transmission of the H1d^FLAG^ allele. H1c^+/−^H1d^+/FLAG^H1e^+/−^ mice were intercrossed to generate H1c^−/−^H1d^FLAG/FLAG^H1e^−/−^ homozygous mice (designated as H1d^FLAG/FLAG^ mice) (Figure S1Ai). These homozygotes were viable, fertile and developed normally as H1c/H1e double null (ce^KO^) mice, demonstrating that FLAG-H1d can substitute for the endogenous H1d to fully rescue the lethal phenotype of H1 TKO mutants. HPLC, mass spectrometry and immunoblotting demonstrated that H1d^FLAG/FLAG^ mice had full replacement of H1d by FLAG-H1d (Figure S1Aii and S1Aiii) and that the H1/nuc ratio of spleen chromatin from H1d^FLAG/FLAG^ mice was 0.7, comparable to that of ce^KO^ mice (Figure S1Aiv). Taken together, these results demonstrate that FLAG-H1d maintains the expression level and properties of the endogenous H1d *in vivo*.

Using a similar knock-in strategy, we generated H1c^+/Myc^H1d^+/−^H1e^+/−^ ESCs (H1c^Myc^) by transfection of the H1c^Myc^ targeting construct into the *cis* triply targeted H1c^+/−^H1d^+/−^H1e^+/−^ ESCs and selected ESC clones that underwent homologous recombination at H1c locus (Figure S1Bi and S1Bii). Similar to FLAG-H1d, the N-terminally Myc tagged H1c (Myc-H1c) colocalized with Hoechst stained nuclear regions in H1c^Myc^ cells (data not shown), and Myc-H1c was eluted in the same fraction as the endogenous H1c protein from HPLC analysis (Figure S1Biii and S1Biv). H1c^Myc^ cells had a H1/nuc ratio of 0.38, comparable to the ratio of 0.36 in ce^het^ cells (Figure 1E, Figure S1Biii), indicating that like FLAG-H1d, Myc-H1c has the same expression level and biochemical properties as the endogenous H1c.

### H1d and H1c are under-represented at GC-, gene-rich regions and depleted at active promoters

**Figure 2:**
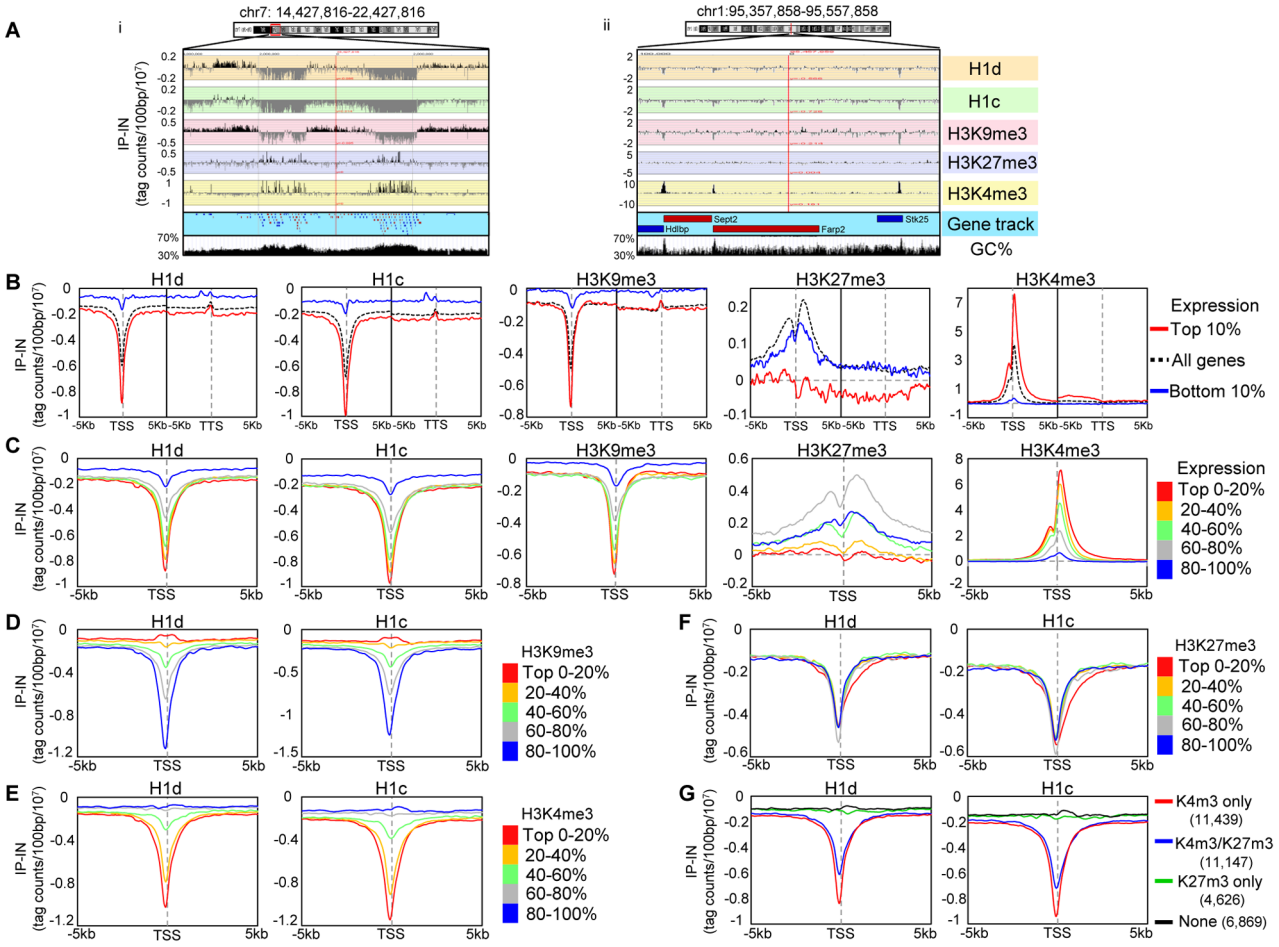
H1 is depleted at GC-rich, gene-rich regions and TSSs of active promoters. (A) Examples of distributions of H1 variants and histone marks at an 8 Mb- (i) and a 200 kb- (ii) region. The GC density track was obtained from UCSC genome browser. Genes are color coded according to their transcription directions (Red: sense strand; Blue: anti-sense strand). (B–C) Metagene analysis of H1d, H1c, H3K9me3, H3K27me3 and H3K4me3 in relation to gene expression levels. TSS: transcription start site. TTS: transcription termination site. B) Profiles of highly active genes (top 10% in expression), silent genes (bottom 10% in expression) and all genes on a 10 kb window around TSS and a 10 kb window around TTS. C) Profiles of genes finely grouped according to expression levels on a 10 kb window centered on TSSs. (D–G) Metagene analysis of H1d and H1c in relation to the levels of H3K9me3 (D), H3K4me3 (E), H3K27me3 (F), and the presence or absence of H3K4me3 and H3K27me3 (G), on regions covering −5 kb to +5 kb of TSS. The number of genes selected within each group in (G) is shown in parentheses. Y axis: tag counts per 100 bp window per 10 million mappable reads. IP-IN: normalized signal values of ChIP-seq subtracted by that of input-seq.

To achieve high resolution mapping of H1d and H1c variants in mouse ESC genome, we performed ChIP-seq in *cis*-targeted H1d^FLAG^ and H1c^Myc^ ESCs using anti-FLAG and anti-Myc antibodies, respectively. In each ChIP-seq library, approximately 80–90% of reads were mappable to the mouse genome (mm9) using the Bowtie aligner [31] (Table S1). While sonicated chromatin input control libraries on average had 65% *vs.* 22% of reads mapped to unique positions and multiple positions respectively, the H1c ChIP-seq libraries had 44% *vs.* 45% mapped to unique *vs.* multiple positions, suggesting that a higher proportion of H1c resides on repetitive sequences. Similarly, an overrepresentation of multi-match sequence reads (39% of mapped reads) occurred in H1d ChIP-seq libraries. A survey of sequencing signal intensities indicated that H1d and H1c were generally depleted from gene rich regions with the deepest dips around transcription start sites of active genes (examples shown in Figure 2A and Figure S2A). ChIP-seq with the anti-FLAG antibody in control ESCs not containing FLAG-H1d generated minimal random background signals (data not shown), and examination of H1c (anti-Myc) signals showed no enrichment at c-Myc target genes, such as *Oct4*, *Nanog* and *Sox2* [32] (Figure S2A), indicating no cross-reactivity for these antibodies. To compare H1 occupancy with other histone marks, we performed ChIP-seq of an active histone mark, H3K4me3, and two repressive histone marks, H3K9me3 and H3K27me3, in murine ESCs. Visual examination of the track files revealed that H1 dips often coincided with H3K9me3 dips or H3K4me3 peaks and that H1 displayed higher signals at gene poor regions with high AT% (low GC%) (Figure 2A). H3K27me3, enriched at *Hox* gene clusters (Figure S2B) as expected, did not show obvious pattern correlation with H1 (Figure 2A and Figure S2A). These observations suggest possible correlations of H1d and H1c with H3K9me3, H3K4me3, gene distribution and GC content *in vivo*.

We next investigated the relationship between H1 occupancy and gene expression levels at a 10 kb region centered around transcription start sites (TSSs) as well as a 10 kb region centered around transcription termination sites (TTSs) using GenPlay software [33]. Such metagene analysis revealed that H1 signals were always lower than chromatin input control within these regions (IP-IN<0) (Figure 2B), suggesting a general depletion of H1 at gene containing regions. Both H1d and H1c were especially depleted around the TSSs with dips much deeper at highly active genes than at silent genes (Figure 2B). Interestingly, except at TSSs and promoters, H1 signals remained largely constant throughout the gene encompassing regions and the signal intensity was higher at the silent genes than that at active genes, suggesting that H1 is underrepresented at surrounding regions of active genes as well (Figure 2B). Indeed, for genes highly depleted of H1 variants at promoters, the signal values of H1s, although gradually increased toward distal regions, remained diminished up to 200 kb from TSS (Figure S3), suggesting that H1s are depleted from broad domains at these regions in the genome. H3K4me3 is known to be peaked around TSS of active genes [34], [35], and metagene H3K4me3 curves displayed an opposite pattern to that of H1 (Figure 2B), further indicating that H1 is absent at active promoters. H3K9me3 exhibited a very similar distribution pattern to that of H1d and H1c, whereas H3K27me3 did not show similar profiles to that of H1 variants (Figure 2B). Metagene analysis of H1 and histone marks on genes finely partitioned by expression levels (each group with 20% of genes) over a 10 kb region (−5 kb to +5 kb of TSS) further corroborated their distinctive patterns at TSSs as a function of gene expression (Figure 2C).

To better define the correlation of H1 occupancy with histone marks around TSSs and promoters, metagene analysis of H1 signals was performed for genes partitioned into 5 groups according to their levels of H3K9me3, H3K4me3, or H3K27me3, which displayed characteristic profiles around TSS (Figure 2D, 2E, 2F and Figure S2C). H1 signals displayed positive and negative correlations with respective H3K9me3 and H3K4me3 signals, having the deepest dip for promoters and TSSs with the lowest H3K9me3 levels (Figure 2D) or highest H3K4me3 signals (Figure 2E). On the other hand, H1 signals showed no correlation with H3K27me3 levels and no difference among the 5 groups of genes partitioned according to H3K27me3 levels (Figure 2F). Interestingly, H1 was also depleted at the promoters of genes bound by H3K4me3 and H3K27me3 bivalent marks [21] but not at H3K4me3-free promoters, regardless of the presence or absence of H3K27me3 (Figure 2G).

Although most H1d and H1c signals appeared universally distributed, we identified regions enriched for H1 binding using SICER [36] and GenPlay software. Identified H1d and H1c enriched regions often formed broad domains (examples shown in Figure S4A). Annotation of H1d- and H1c- rich regions using CEAS [37], a software designed to characterize both sharp and broad ChIP-seq enrichment, indicated that, similar to H3K9me3, both H1d and H1c “peaks” were over-represented in distal intergenic regions and under-represented at promoters and 5′UTR, which were highly enriched with H3K4me3 peaks as reported previously (Figure S4B and [34]).

### Correlation of H1 with histone marks and features of H1d and H1c enriched regions

**Figure 3:**
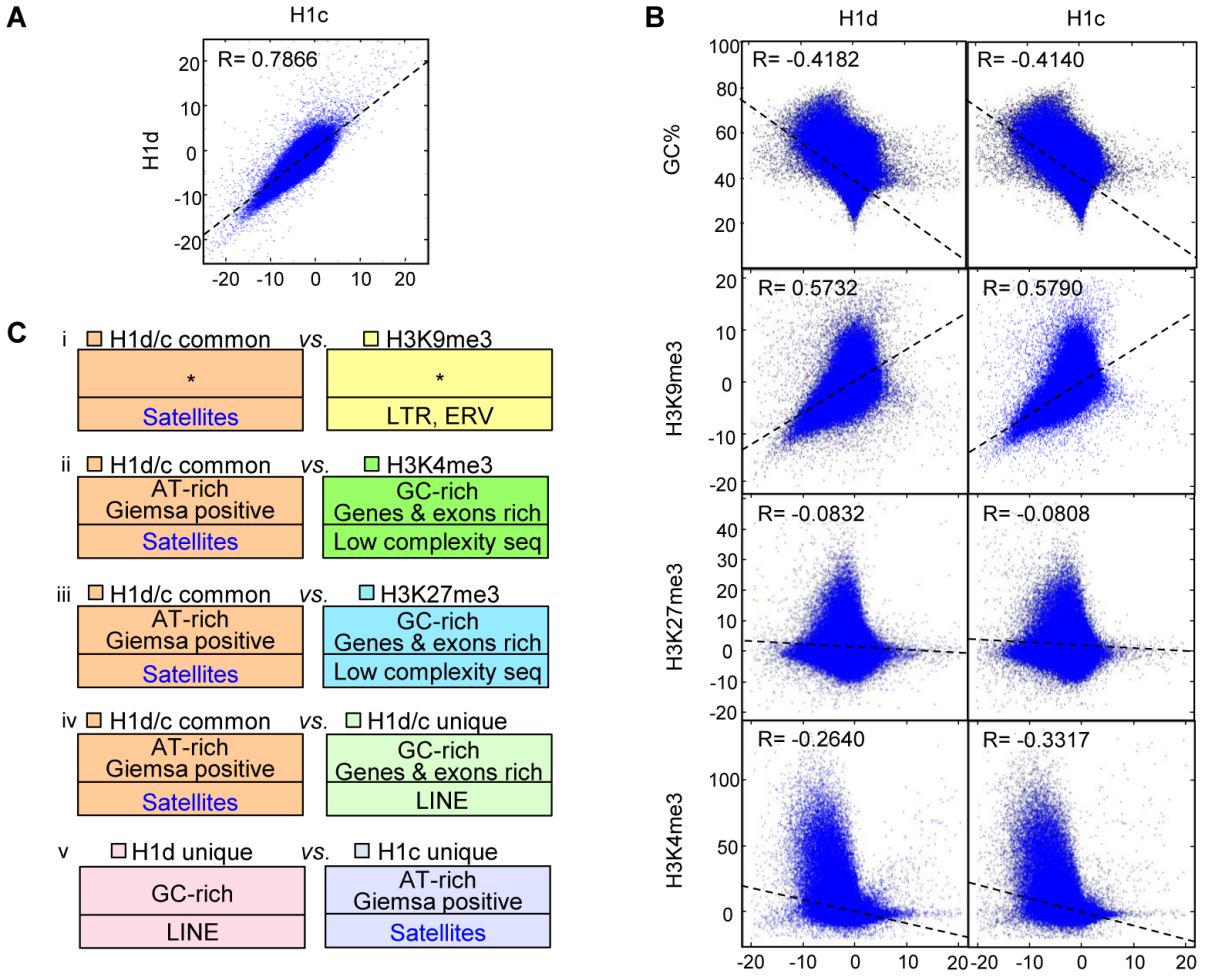
Correlation and over-representation analyses of H1 variants and histone marks. (A–B) Genome-wide correlation scatter plots of H1d *vs.* H1c (A), and GC% (or histone marks) *vs.* H1d (left) and H1c (right) (B). The correlation coefficient and the trend line were generated as described in methods. X and Y axes: average signal values (normalized to 100 bp window). Pearson's correlation was used to perform the analysis. P<10^−100^ for all correlation coefficient. (C) Overrepresented features from the following 5 comparisons of H1 or histone mark highly enriched regions; i) H1d/H1c common *vs.* H3K9me3 regions; ii) H1d/H1c common *vs.* H3K4me3 regions; iii) H1d/H1c common *vs.* H3K27me3 regions; iv) H1d/H1c common *vs.* H1d/H1c unique regions; v) H1d unique *vs.* H1c unique regions. Bottom half of each box: repetitive elements. *: no significant overrepresentation. All P values remained significant after multiple testing corrections with the FDR method and the more conservative Bonferroni method.

We next performed genome-wide correlation analysis to determine if the similarity and/or contrast of H1 variants with GC% and histone marks at TSSs also extend to a genome-wide scale. Indeed, the distribution of H1d and H1c were highly correlated throughout the genome (R = 0.7866) (Figure 3A), and both variants were negatively correlated with GC% (R = −0.4182 and −0.4140 for respective H1d and H1c), indicating that H1d and H1c were enriched or depleted at similar regions. Both H1d and H1c were correlated negatively with H3K4me3 (R = −0.2640 and −0.3317 respectively), but positively with H3K9me3 (R = 0.5732, 0.5790) (Figure 3B), suggesting their enrichment at heterochromatin. On the other hand, these two variants showed no obvious correlation with H3K27me3 (R = −0.08 for both variants) (Figure 3B). Correlation analysis of sequencing signals on enriched or depleted regions gave similar coefficients as the respective genome-wide coefficients (data not shown). It is interesting to note that the coefficients of H1 *vs.* H3K4me3 on sex chromosomes were dramatically different from those of autosomes (Figure S5A). This result echoes the previous finding that sex-chromosome genes are overrepresented among genes with altered expression levels by triple H1 deletion in ESCs [26], suggesting that H1 may play a role in regulating higher order chromatin structures of sex chromosomes.

To gain a comprehensive view of the DNA features of H1d- and H1c- rich regions, we selected the regions highly enriched for H1 variants and histone marks, and performed cross-comparison of genome attributes using the statistical analysis software EpiGRAPH [38]. Such analysis (Figure 3C and Figure S5B) revealed that: *a*) H1d/H1c common peaks (regions highly enriched for both H1d and H1c) appeared similar to H3K9me3 peaks in genome attributes, except for satellite DNA which was relatively overrepresented in H1 peak regions; *b*) H1d/H1c common peaks were enriched at AT-rich sequences, satellite DNA, and chromosome G-bands but were absent from GC-rich regions, and genes or exons when compared with H3K4me3 or H3K27me3 peaks; *c*) comparison of H1d/H1c common peaks with H1d/H1c unique peaks (regions highly enriched for H1d or H1c but not both) showed similar features as the comparison of H1d/H1c common peaks with H3K4me3 or H3K27me3 peaks; *d*) comparison of H1d *vs.* H1c specific peaks indicated that H1d unique peaks were relatively enriched at GC-rich sequences and LINEs, whereas H1c unique peaks were more enriched at AT-rich sequences, Giemsa positive regions and satellite DNA; *e*) the overrepresentation analyses between H1d (or H1c) unique peak regions and histone mark peak regions exhibited similar features as comparisons using H1 common peaks. These results define common and unique features for H1d and H1c enriched regions.

### High occupancy of H1d and H1c at major satellite sequences

**Figure 4:**
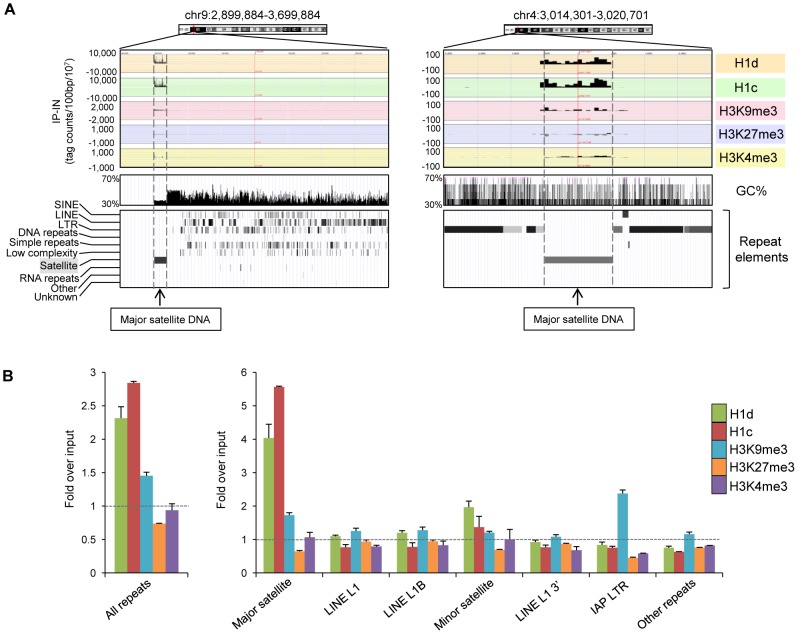
H1d and H1c are enriched at the major satellite sequences. (A) Representative profiles of top H1d and H1c enriched regions (mapped to mm9). Repeat element tracks were obtained from UCSC genome browser. Dashed lines indicate the localization of these H1 peaks at major satellite sequences. (B) Fold enrichment of percent mappable repeats (mapped to RepBase) from H1d, H1c, and histone marks ChIP-seq libraries over that from corresponding chromatin input-seq library on all repeats (left), six most abundant repetitive sequences and the remaining other repeats (right). The dashed lines indicate the level of normalized input signal. P values calculated with Fisher's exact test comparing ChIP-seq with input-seq libraries are less than 2.5×10^−5^ for all repeat classes shown. Error bars represent the differences between replicates. Data are presented as average ± S.E.M.

The EpiGRAPH overrepresentation analysis indicated that peak regions of H1d and H1c were enriched for satellite repeats. Indeed, examination of the top ranked H1 peak regions with especially high binding signals revealed that these regions overlap perfectly with major satellite sequences (examples shown in Figure 4A). This finding and the above observation of overrepresentation of multi-match sequence reads in H1 ChIP-seq libraries prompted us to perform a thorough mapping study of sequence reads to a database of repetitive sequences. We aligned sequence reads of H1d, H1c, H3K9me3, H3K27me3 and H3K4me3 ChIP-seq libraries to Repbase Update, a comprehensive database of repetitive elements from diverse eukaryotic organisms [39]–[41]. We found that both H1d and H1c were significantly enriched at repetitive sequences, with H1d and H1c ChIP-seq libraries having on average percent mapped repeats respective 2.3-, and 2.8-fold of that of chromatin input-seq libraries (Figure 4B). H3K9me3, H3K27me3 and H3K4me3 ChIP-seq libraries had an average respective percent mapped repeats 1.4-, 0.7-, and 0.9- fold compared with input controls (Figure 4B), suggesting an overrepresentation of H3K9me3, yet not as dramatic as H1d and H1c, at repetitive sequences.

Importantly, we found that the increased proportion of total reads of H1 libraries mapped to repetitive sequences was predominantly caused by overrepresentation on the major satellite sequences on which the levels of H1d and H1c occupancy were enriched on average 4.0- and 5.6-fold compared with the chromatin input control (Figure 4B). This level of H1 enrichment appeared to be specific to major satellites because we did not observe H1d and H1c enrichment among other abundant repeats, except for a moderate increase of H1d and H1c occupancy at minor satellites. qChIP-PCR results confirmed the preferential binding of these two H1 variants to major satellites (Figure S6). Sequencing results showed that H1d and H1c levels on most of other less abundant classes of repetitive elements, such as L1, IAP LTR retrotransposons, SINE, non-LTR retrotransposons, and DNA transposons, were similar or lower compared with the input control (Figure 4B and Figure S7). H3K4me3 was highly enriched at 5′end of a subset of LINE L1 sequence (Figure S7), consistent with the abundant expression of L1 detected in multiple cell types [42]–[44], whereas H3K9me3 was enriched at major satellite repeats and LTR transposons, such as IAP particles, with similar levels as previously reported [34], [45] (Figure 4B and Figure S7). Enrichment of H1 variants at major satellites was also confirmed by calculating the normalized “IP-IN” signals at major satellite regions in mouse genome mm9 assembly (July 2007) annotated by RepeatMasker (http://repeatmasker.org) (Figure S8). Analysis of ChIP-seq libraries of FLAG-H1d in H1d-*trans* ESCs, which had similar levels of FLAG-H1d and total H1/nuc ratio as the *cis* H1d^FLAG^ ESCs, also showed similar level of enrichment at major satellites as H1d^FLAG^ ESCs (Figure S9).

### Increased nucleosome repeat length at major satellite sequences in ESCs

**Figure 5:**
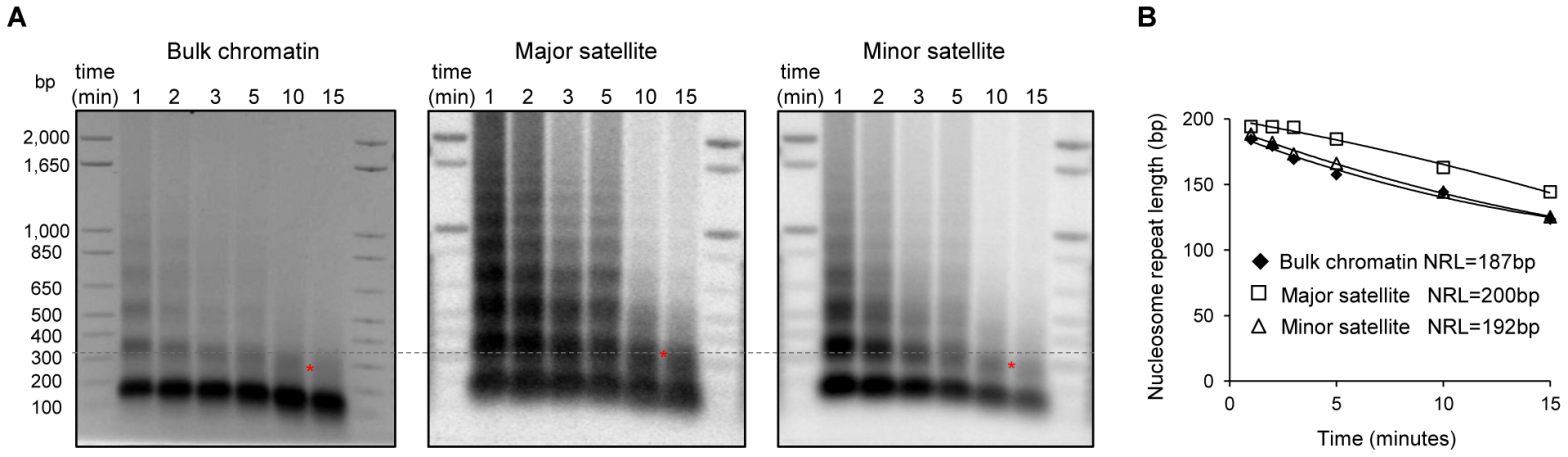
Increased nucleosome repeat length at major satellite repeats in ESCs. (A) Nucleosome repeat length analyses of bulk chromatin (left), major satellite sequences (middle) and minor satellite sequences (right) in WT ESCs. DNA isolated from ESC nuclei digested with MNase at different time points were analyzed by ethidium bromide (EB) –stained gel (left), transferred to membrane which was sequentially probed with major satellites (middle) and minor satellites (right) using Southern blotting. The positions of di-nucleosomes with 10-minute MNase digestion are marked by *. The dashed line indicates di-nucleosome position of major satellites, which is higher than that of bulk chromatin and minor satellites. (B) The NRLs were calculated from the images presented in (A) by extrapolating the corresponding curves to time “0” as described [72].

The level of H1 has been shown to be a determinant of nucleosome repeat length (NRL) with a higher level of H1 correlating with a longer NRL [46], [47]. To validate the enrichment of H1 variants at major satellites and to investigate its impact on the local chromatin structure at these regions, we measured the NRL of bulk chromatin and that of the pericentromeric (major satellites) and centromeric (minor satellites) regions with a time-course micrococcal nuclease (MNase) digestion assay. Southern blotting images revealed that chromatin at major satellites was more resistant to MNase digestion than bulk chromatin and minor satellites (Figure 5A). Consistent with previous studies [26], the bulk chromatin of mouse ESCs displayed a NRL of ∼187 bp (Figure 5B). However, the NRL at major satellites had a value of 200 bp, which was ∼13 bp and ∼8 bp longer than the NRLs of respective bulk chromatin and minor satellites in ESCs (Figure 5B). These results suggest that the enrichment of H1d and H1c at major satellite repeats may contribute to the increase of NRL in the pericentromeric region compared with bulk ESC chromatin. Analysis of H1c/H1d/H1e triple knockout (H1 TKO) ESCs established previously, which have an H1/nuc ratio of 0.25 in bulk chromatin compared with that of 0.46 in WT ESCs [26], indicated that H1 depletion caused a proportional decrease of NRLs in bulk chromatin, major satellites and minor satellites (Figure S10). Consistently, qChIP analysis using a pan-H1 antibody showed total H1 levels were reduced at major and minor satellites by H1 depletion (Figure S10D).

### H1 depletion leads to chromocenter clustering and de-repression of major satellite repeats independent of multiple epigenetic marks

**Figure 6:**
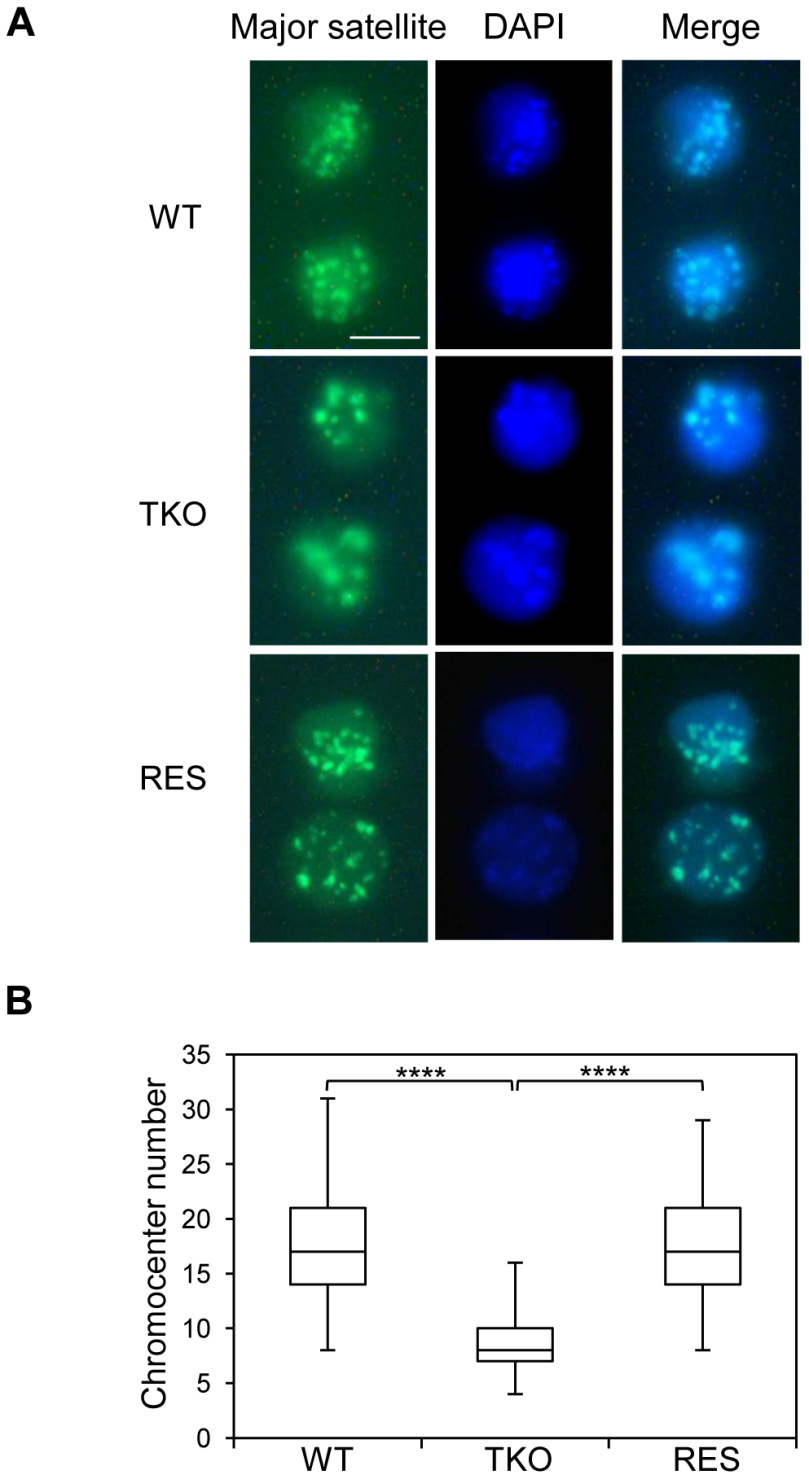
H1 depletion leads to chromocenter clustering. (A) Typical images of WT (top), H1 TKO (middle), and RES ESCs (bottom) of FISH with a major satellite probe (left), DNA stain DAPI (middle), and merged images (right). Scale bar: 10 µm. (B) Box plots of chromocenter numbers in the nuclei of WT, H1 TKO, and RES ESCs. The line in the box indicates the median, while the bottom and top of the boxes are the 25^th^ and 75^th^ percentiles, respectively. ****: P<0.000001.

Major satellite repeats at pericentric heterochromatin from different chromosomes tend to cluster together and form the chromocenter, a nuclear compartment that plays an important role in structural maintenance of the chromosomes [48], [49]. Several chromatin proteins such as MeCP2, MBD2, DNMT3a, DNMT3b, and UHRF1 have been shown to contribute to chromocenter clustering [50]–[52], however, the role of H1 in chromocenter formation has not been studied to date. Since both H1d and H1c are markedly enriched at major satellites, we set out to determine the effects of H1 depletion on chromocenter clustering in WT and H1 TKO ESCs by fluorescence in situ hybridization (FISH) using a major satellite specific probe. The chromocenter numbers in H1 TKO ESCs (median = 8, n = 160) were significantly lower than WT cells (median = 17, n = 206) (Figure 6), and the size of chromocenters in H1 TKO ESCs on average was bigger than that in WT ESCs (Figure S11), demonstrating a previously unnoticed defect in the pericentromeric chromatin structure caused by H1 depletion. Analysis of “rescue” (RES) cells established previously [53] showed that overexpressing H1d in H1 TKO cells effectively restored the size and the numbers of chromocenters to the levels comparable to WT cells (Figure 6 and Figure S11). Similarly, H1d^FLAG^ and H1c^Myc^ cells displayed normal chromocenter clustering as WT ESCs (Figure S15). These results indicate that the increased chromocenter clustering is likely due to the dramatic decrease of total H1 levels in H1 TKO ESCs.

**Figure 7:**
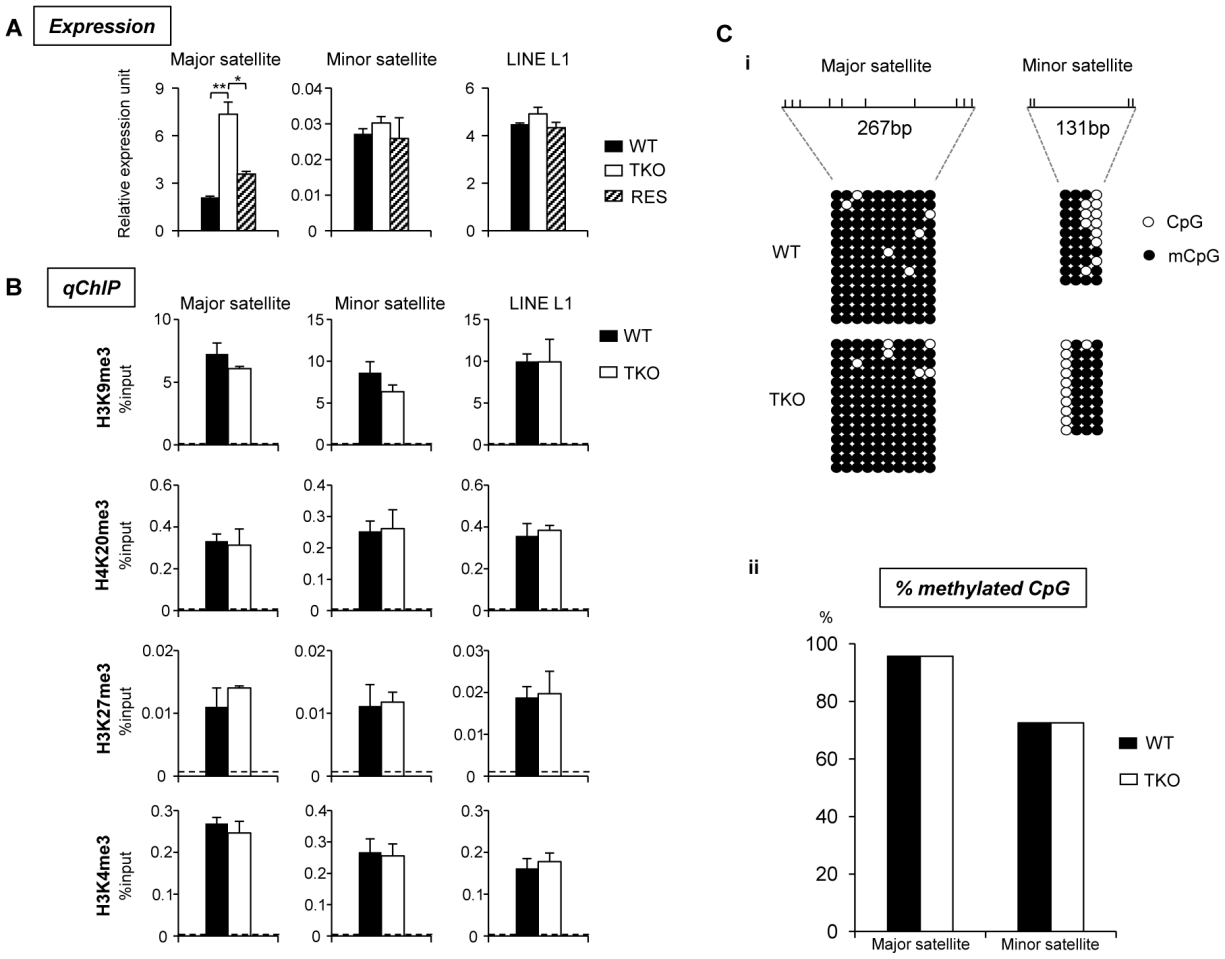
H1 depletion leads to increased expression of major satellite repeats independent of multiple epigenetic marks. (A) Analyses of expression of selected repeats in WT, H1 TKO, and RES ESCs by qRT-PCR. Data are represented as mean +/− S. D.. *: P<0.05; **: P<0.01. (B) qChIP analysis of three repressive histone marks and one active histone mark at selected repetitive sequences in WT and H1 TKO ESCs. Dashed lines indicate the highest level of signals detected by qChIP with IgG antibody. (C) Bisulfite sequencing analysis (i) and percent of methylated CpG (ii) of major, minor satellite sequences. The positions of CpG sites analyzed are marked as vertical ticks on the line.

Pervasive transcription of repetitive sequences contributes to genome regulation, and aberrant regulation of the expression of satellite sequences interferes with heterochromatin assembly and chromosome segregation [49], [54]–[56]. To further examine the effects of H1 depletion on major satellites, we analyzed several repetitive sequences for expression and epigenetic marks in WT and H1 TKO ESCs. Quantitative reverse transcription-PCR (qRT-PCR) analysis showed that the expression levels of major satellites were 3.5-fold higher in H1 TKO ESCs than in WT ESCs, whereas the expression levels of minor satellites and LINE L1 were not significantly changed (Figure 7A). Such de-repression of major satellites by H1 depletion was dramatically curbed in RES cells (Figure 7A) as well as in H1d^FLAG^ and H1c^Myc^ ESCs (Figure S16), indicating that the levels of H1s have a direct impact on transcriptional regulation of major satellites. Notably, the levels of multiple epigenetic marks, such as repressive marks H3K9me3, H3K27me3, and H4K20me3, the active mark H3K4me3, as well as DNA methylation all remained unchanged at the analyzed repeats in H1 TKO ESCs compared with WT ESCs (Figure 7B and 7C). The lack of significant changes in the histone marks and DNA methylation at these repetitive sequences suggests that the increase in expression levels at major satellites may be due to an effect of local chromatin decondensation caused by H1 depletion in H1 TKO ESCs.

We note that the level of H1^0^, the replacement H1 variant, was increased significantly in TKO ESCs compared with that in undifferentiated WT ESCs where H1^0^ was minimal [26], [53]. To examine if the increased chromocenter clustering and expression of major satellites in H1 TKO ESCs could be attributed to an increase in H1^0^ levels, we generated “fH1^0^” cells by over-expressing FLAG-H1^0^ in WT ESCs, and selected cell lines that expressed FLAG-H1^0^ at a similar level to that of H1^0^ in H1 TKO ESCs (Figure S12 and [26]). As expected, FLAG-H1^0^ was eluted in the same fraction as endogenous H1^0^. ChIP-seq of H1^0^ in fH1^0^ cells with an anti-FLAG antibody indicated that, despite its different biochemical properties and unique expression patterns [6], [8], [57], H1^0^ shared similar distribution features to that of H1d and H1c in ESCs, including depletion at active promoters and enrichment at major satellites (Figure S3, Figure S8, Figure S13, and Figure S14). Similar to H1d and H1c, H1^0^ also displayed overall positive correlation with H3K9me3 and inverse correlations with GC% and H3K4me3, although the level of correlation was to a lesser extent (data not shown). Furthermore, H1^0^ enriched regions were significantly under-represented in gene regions but over-represented in distal intergenic regions with 80.1% of H1^0^ peaks located in these regions (data not shown). Beside major satellites, H1^0^ also appeared to be enriched at minor satellites and, to a lesser extent, at LINE L1 elements as determined by ChIP-seq and ChIP-PCR (Figure S14B and S14C), suggesting differential binding preferences of H1^0^ compared with H1d and H1c.

Analysis of fH1^0^ ESCs by FISH and qRT-PCR indicated that the chromocenter numbers were not reduced compared with WT ESCs (Figure S15) and that expression of major satellites remained at low levels (Figure S16), excluding the possibility of H1^0^ upregulation being responsible for chromocenter clustering and upregulation of major satellite transcription in H1 TKO ESCs.

Collectively, these results demonstrate increased chromocenter clustering and major satellite transcription by H1 depletion, and suggest important roles of the dominant H1 variants in ESCs in maintaining pericentric chromatin properties.

## Discussion

H1 Linker histones are abundant chromatin binding proteins that facilitate the formation of higher order chromatin structures [1], [2]. The existence of multiple mammalian H1 variants which are differentially regulated during development presumably offers additional levels of modulation on chromatin structure and function. Despite many efforts, the *in vivo* localization and function of individual H1 variants in genome organization remain elusive. Chromatin plays critical roles in stem cell fate determination and reprogramming, and the epigenome of ESCs has been intensively studied. However, the genome-wide maps of one group of the major chromatin proteins, H1 variants, have not been established. Here, we have filled both gaps by generating high resolution maps of three H1 variants in mouse ESCs, identified unique H1 binding features, and discovered an unusual enrichment and function of H1 variants at major satellites.

We have established a knock-in system to stringently test the functions of the tagged H1s and to facilitate the generation of high resolution maps of H1 variants in ESCs by ChIP-seq. Our results demonstrate that, when tagged at the N-terminus, the short FLAG and Myc tags, with respective 8 and 13 amino acids, do not alter the biochemical and cellular properties of H1 proteins *in vivo*. The strategy of homologous recombination ensures that the expression of tagged H1 variants is comparable to that of their endogenous counterparts. FLAG-H1d fully rescues the lethal phenotype of H1d deletion on H1c/H1e double knockout genetic background, further demonstrating the functional equivalence of the tagged H1 and the respective endogenous H1 variant *in vivo*. Although Myc-H1c was not tested in mice, it is anticipated to mimic the endogenous H1c based on all the other assays performed. These data provide a technical demonstration on how highly similar protein variants can be analyzed differentially and on a genomic scale using *in vivo* validated knock-in mice.

On the H1 genome-wide maps we have generated here, H1d and H1c are highly correlated and display similar binding patterns in the ESC genome. Both variants are enriched at AT-rich regions, gene deserts and major satellites, but are depleted at GC-rich, gene-rich regions and especially at active promoters. Thus, despite their differences in compacting DNA *in vitro* and the expression patterns during development [8], [10], [28], H1d and H1c are quite similar in overall distribution in the genome, which we surmise contributes to the redundancy among the major somatic H1s as suggested from previous studies of single or double H1 variants knockout mice [7], [14]. Nevertheless, analyses of the regions that are uniquely enriched for H1d or H1c reveal some differences in sequence features (Figure 3C and Figure S5B). H1c has a higher enrichment at major satellites than H1d but is relatively depleted from LINE sequences (Figure 3C and Figure 4B). In addition, H1c enriched regions have a higher proportion in gene bodies and proximal regions compared with H1d peak distribution (Figure S4B). These differences may account for an additional level of modulation and fine-tuning of genome function by the presence of multiple H1 variants in mammals.

H1^0^, the H1 variant associated with differentiation, has unique expression pattern and biochemical properties. It is highly basic, expressed in differentiated cell types, and more similar to histone H5 in avian red blood cells than any other somatic variants [53], [57]. However, overexpressed H1^0^ (in fH1^0^ cells) shares the distinctive features of H1d and H1c in ESCs in genome-wide occupancy. It is worth noting, though, that endogenous H1^0^ proteins are present at very low levels in undifferentiated WT ESCs and the genome-wide localization of H1^0^ in ESCs may differ significantly from its binding patterns in differentiated cells. It would be interesting to systematically determine the genome-wide maps of histone variants in different cell types, particularly in light of a recent study reporting a distribution pattern change of H1.5 in cellular differentiation [58]. The cell lines and mouse models generated in this study will greatly facilitate these future studies.

The prevalent H1 variants binding with local troughs at active promoters we observed here in the mouse ESC genome is reminiscent of the previous results when ChIP-chip and a pan-H1 antibody were used to map H1 on a portion of the human genome in MCF-7 cells [59] or when DamID method was used to map H1 in *Drosophila* cells [60]. The depletion of H1 at TSSs of active genes observed in three systems suggests that this feature is common to all H1s and evolutionarily conserved. However, our study differs from the two previous studies and offers more opportunities for high resolution and in-depth analysis because the knock-in system generated in this study allows for robust and highly specific mapping of H1 variants and deep-sequencing covers the entire genome including the repetitive genome. Furthermore, we have found that the depletion of H1 at active genes is not restricted to regions around the TSS, but also expands to the entire gene encompassing domain (Figure 2B and 2C). Such phenomena suggests that a wide-spread change in higher order chromatin structure may be associated with gene expression and that gene-rich domains may adopt an overall decondensed chromatin structure with less H1 occupancy.

Correlation analyses indicate that H1d and H1c are inversely correlated with GC content, H3K4me3 mark, but positively correlated with H3K9me3 mark across the mouse ESC genome (Figure 3B). Our finding that the common peaks of H1d and H1c are enriched with AT-rich DNA sequences *in vivo* resonates with the previous observation that H1 is preferentially associated with scaffold associated regions (SAR) [61], which are also AT-rich sequences [62]. This binding feature may reflect a higher affinity of H1 to AT-tracts observed in *in vitro* studies [63], [64]. The GC content has been suggested to be an intrinsic factor for nucleosome occupancy [65], and our data suggest that it may also have an impact on H1 binding. It is also noteworthy that, compared with gene expression levels, H3K4me3 and H3K9me3 correlate better with H1 levels at TSS. For example, we did not observe dips of H1d and H1c around promoters of 40% genes when partitioned by H3K4me3 or H3K9me3 signals, whereas a small H1 signal dip exists even for the 20% genes with lowest expression values (Figure 2C, 2D, and 2E). It is possible that the steady state level of RNA messages (expression) may not faithfully reflect the active/inactive state of the promoters which may correlate better with the status of histone marks. It has been reported that promoters of many genes with low expression have high H3K4me3 levels [21], and we surmise that H1 may be absent from these gene promoters as well.

The co-localization of H1d and H1c with H3K9me3 suggests that these two variants are enriched at heterochromatin and may facilitate the maintenance of constitutive heterochromatin structure. Such association may be mediated through HP1, the heterochromatin protein binding to H3K9me3 and H3K9 methyltransferase Suv39h and facilitating spreading of heterochromatin marks [66]–[68]. Indeed, H1 has been shown to interact *in vitro* with HP1α [69], [70]. On the other hand, localization of HP1 is impaired in H1 depleted *Drosophila* [71], suggesting that H1 may also contribute to the proper targeting of HP1.

Surprisingly, we found that, at major satellite sequences, H1d and H1c signals are dramatically overrepresented, and this accounts for almost all the increased proportion of H1 sequence reads at repetitive sequences. The levels of H1d and H1c at major satellites are much higher than H3K9me3 (Figure 4B), a repressive histone mark also enriched at these repeats [34]. The overrepresentation of H1 at major satellites in ESCs is also supported by a longer NRL, which suggests a higher local H1 level than bulk chromatin and minor satellites. Consistent with previous observations [49], [51], we find that major satellites are more resistant to MNase digestion than bulk chromatin and minor satellites in ESCs (Figure 5), suggesting that pericentromeric regions may adopt special higher order chromatin structure as indicated by sucrose sedimentation assay [72]. High resolution mapping in this study identifies major satellites as the dominant preferential binding sites for H1 variants in ESCs, suggesting that H1 may play an important role in mediating the formation of distinct chromatin structure at pericetromeric regions. This is further supported by the effects of H1 depletion on chromocenter clustering and expression of major satellites. We note that a higher NRL in major satellites than bulk chromatin is also present in H1 TKO ESCs (Figure S10), suggesting a possible enrichment of the remaining H1 variants at major satellite sequences in H1 TKO ESCs. Consistently, we find that overexpressed H1^0^ also appear to preferentially accumulate at satellite sequences in ESCs (Figure S14).

The enrichment of H1 at major satellites could not be solely attributed to the relatively high affinity of H1c and H1d to AT-rich sequences. Major and minor satellites sequences contain approximately 65% of A and T, with a ratio of A∶T being respective 2.6∶1 and 1.8∶1. This could result in major satellites having more A-tracts to which H1 might have a higher affinity. Phased nucleosome positioning observed at the major satellites [73], [74] could also contribute to the preferential binding of H1 at this region because different nucleosome positioning patterns have been shown to differentially affect H1 binding *in vitro* [75].

Mouse major satellites, constituting the pericentromere [76], [77] necessary for chromosome structure and function, are shown to form clusters/chromocenters, exhibit distinct heterochromatin features and adopt a more stable and condensed chromatin conformation than the bulk chromatin [49], [72]. Our findings of the preferential binding of H1 at major satellites and chromocenter clustering (reduced number of chromocenters) in H1 TKO ESCs suggest that H1 contributes to and may be required for the proper formation of pericentric heterochromatin. The rescue of the clustering effects by overexpressing H1d in H1 TKO ESCs or in H1d^FLAG^ and H1c^Myc^ cells compared with H1 TKO ESCs indicates that the total H1 level, rather than a specific H1 variant, is a key determining factor of chromocenter clustering. This conclusion is further supported by our finding that overexpressing H1^0^ level to 3.5 fold of that of endogenous H1^0^ in WT ESCs has little effect on chromocenter numbers or major satellite expression. *In vitro* studies have shown highly cooperative binding of H1 globular domain to DNA [78], a property which we speculate could contribute to increased chromocenter clustering in the face of marked reduction of H1 levels in H1 TKO ESCs. A larger nucleosome spacing (200 bp) (Figure 5) together with a higher local H1 level at major satellites could be important for efficient compaction of pericentromeric chromatin because nucleosome arrays with a NRL of 197 bp are able to form 30 nm fiber structure *in vitro* in the presence of linker histones whereas arrays with a short NRL are only able to form thinner and less compact structures [5].

The effects of H1 on major satellites are not restricted to chromatin structure and heterochromatin formation. Loss of H1c, H1d and H1e causes a dramatic increase in transcripts from major satellites, but does not change the levels of the repressive epigenetic marks, H3K9me3, H4K20me3, H3K27me3, or DNA methylation at these sequences. This suggests that the increase in expression of major satellites in H1 TKO ESCs is not mediated by loss of these repressive epigenetic marks, but rather caused by reduced binding of H1 per se or the potential decondensation of local chromatin structure. The phenomenon of changes in chromocenter organization independent of H3K9me3 is reminiscent of results from deletion of UHRF1 [52], a histone binding protein or overexpression of MeCP2 in mouse myoblasts [50]. Chromocenter organization is likely to be independent of H3K9me3 pathway because double deletion of Suv39h1 and Suv39h2 has minimal effects on the number and size of chromocenters in mouse cells [79], [80]. The expression changes in major satellites in H1 TKO ESCs are also not due to potential changes in cell cycle since H1 TKO ESCs have similar growth rate [26] and cell cycle profiles (data not shown) to WT ESCs. The reduction in expression levels of major satellites detected in RES cells compared with H1 TKO cells further supports that the drastic decrease in H1 levels causes de-repression of major satellites. Noncoding major satellite transcripts have been shown to be important for proper chromocenter formation [81], thus we speculate that the increased levels of major satellite transcripts contribute to chromocenter clustering in H1 TKO cells. In light of previous findings that ESCs null for DNA methyltransferases displayed chromocenter clustering [51], similar to what we observed in H1 TKO ESCs, we surmise that H1 and DNA methylation may act cooperatively in the proper maintenance of chromocenter structure.

In summary, we report high resolution maps of two abundant somatic H1 variants and the replacement H1 variant in mouse ESCs, connecting this important yet under-explored repressive mark with the well-studied ESC epigenome. The enrichment and effects of H1d, H1c and H1^0^ on major satellites highlight an important role of these H1 variants in the maintenance of chromosome architecture and function. The cell lines and mouse strains we generated using the knock-in system also provide valuable tools for studying H1 variant specific functions both *in vitro* and *in vivo*. Genome-wide distribution studies of other H1 variants as well as in differentiated cell types are likely to lead to a better understanding of the role of H1 and higher order chromatin folding in gene expression and chromatin function.

## Materials and Methods

### Generation of H1d^FLAG^ ESCs and H1d^FLAG/FLAG^ mice

The H1d^FLAG^ knock-in targeting vector containing H1d 5′ and 3′ homology regions flanking the N-terminal FLAG-tagged H1d and the SV40-Blasticidin resistant gene was transfected into ESCs as described previously [14]. 200 ESC clones resistant to 20 µg/ml Blasticidin (Life Technologies) and 2 µM gancyclovir (Sigma-Aldrich) were picked, and 5 clones with homologous recombination were identified by Southern blotting using the probe shown in Figure 1A. Two *cis*-targeted clones were injected into C57BL/6 recipient blastocysts to produce chimeric mice, which gave germline transmission. H1c^+/−^H1d^+/FLAG^H1e^+/−^ mice were intercrossed to generate H1c^−/−^H1d^FLAG/FLAG^H1e^−/−^ (H1d^FLAG/FLAG^) mice. All animal work was performed according to procedures approved by the Institutional Animal Care and Use Committee (IACUC) at Georgia Institute of Technology.

### Preparation and analysis of histones

Nuclei and chromatin of ESCs and mouse tissues were prepared and analyzed according to protocols described previously [82], [83]. Histones were extracted from chromatin with 0.2 N sulfuric acid and 50–100 µg of total histone preparations were injected into a C18 reverse phase column (Vydac) on an ÄKTA UPC10 system (GE Healthcare). The effluent was monitored at 214 nm (A_214_), and the peak areas were recorded and analyzed with ÄKTA UNICORN 5.11 software. The A_214_ values of the H1 and H2B peaks were adjusted by the number of peptide bonds in each H1 variant and H2B. The H1/nucleosome ratio was determined by dividing the A_214_ of all H1 peaks by half of the A_214_ of the H2B peak. Fractions corresponding to different H1 variants from HPLC analysis were collected, lyophilized and analyzed with silver staining, Coomassie staining and Western blotting.

### Antibodies

The following antibodies were used in this study: anti-FLAG (Sigma-Aldrich F3165), anti-DYKDDDDK tag (Cell Signaling #2368), anti-Myc-tag (Cell Signaling #2272), anti-H3K4me3 (Millipore 07-473), anti-H3K9me3 (Abcam 8898), anti-H3K27me3 (Millipore 07-449), anti-H4K20me3 (Millipore 07-463), anti-H1^0^ (Santa Cruz 56695), anti-H1 (Milipore 05-457) and IgG (Millipore 12-370).

### Chromatin immunoprecipitation (ChIP)

ChIP assays were performed as described previously [26] with the following modifications: 20 µl of Dynabeads Protein G (Life Technologies) were incubated with 2 µg of antibody for 4 hours, followed by incubation with 40 µg of sonicated soluble chromatin overnight at 4°C. Dynabeads were washed, immunoprecipitates were eluted, and DNA-protein complexes were incubated overnight at 65°C to reverse crosslinks. DNA was purified with a DNA Isolation column (Qiagen). Input control DNA was prepared from reverse-crosslinked soluble chromatin prior to immunoprecipitation. Quantitatitve PCR on ChIP samples for major satellites, minor satellites, LINE L1, IAP LTR and Hprt was performed with primers published previously [45], [84].

### Generation of ChIP–seq libraries

The libraries for massive parallel sequencing were prepared with the ChIP-seq Sample Preparation Kit (Illumina) according to the manufacturer's instructions. Briefly, 10 ng of immunoprecipiated DNA or input DNA were end repaired, 3′ adenylated and ligated with adapter oligos supplied by the manufacturer. DNA fragments within the range of 120∼500 bp were purified following gel electrophoresis and amplified with primers provided by the manufacturer. Library DNA was subsequently purified with a Qiagen DNA Isolation column, quantified and submitted for sequencing.

### Sequence reads processing and alignment

Sequencing was performed with Illumina Genome Analyzer II and Illumina HiSeq 2000 systems, and raw sequence reads containing more than 30% of ‘N’ were removed and adaptor sequences were trimmed. Clean sequences were aligned against mouse genome, mm9 (UCSC website), and 2,669 categories of mammalian repeats from RepBase version 14.07 [39], [40] using Bowtie aligner software (http://bowtie-bio.sourceforge.net/index.shtml). The first 40 bp (for alignment to mm9) or the first 35 bp (for alignment to RepBase) of the reads were used as seed sequences with up to two mismatches allowed for the alignment, and aligned number of reads were scored. Reads with multiple alignment positions were mapped randomly to one of the possible position. Reads for each ChIP-seq or input-seq library aligned to mm9 were normalized to 10 million reads, and IP-IN signals were calculated in each 100 bp sliding window by subtraction of normalized read counts per 10 million mappable reads of ChIP-seq library by that of its corresponding input-seq library using GenPlay software (http://genplay.einstein.yu.edu/wiki/index.php/Documentation) [33]). Percentage of reads for each repeat mapped to RepBase was calculated by dividing reads mapped to the respective repeat by the total reads in the library, and the fold enrichment for the respective repeat was subsequently calculated as the ratio of the percent of reads of ChIP-seq library to that of the input-seq library. Read length and read counts of each library are listed in Table S1. Representative ChIP-seq libraries with the most sequencing reads mapped to mm9 were utilized for genome browser visualization and metagene analysis, and all replicate ChIP-seq libraries were included in repetitive sequence analysis. Sequencing data have been deposited in NCBI's Gene Expression Omnibus database and assigned GEO Series accession number GSE46134.

### Genome-wide correlation analysis

The sum of signals (IP-IN) for each 1000 bp window (normalized to 10 million reads) was used to calculate the correlation coefficients of H1 variants with GC% and different histone markers. Genome-wide and chromosome-wide correlation coefficients were calculated, and the scatter-plots were generated using Matlab.

### Overrepresentation and distribution pattern analysis

Significantly enriched regions were identified using GenPlay or SICER v1.1 [36] at the following parameter settings: window size = 200, gap size = 600, E-value = 1000, an effective genome size of 80% of the entire mouse genome, and q-value (FDR) = 0.001. In order to optimize the gap size for H1 variants, the gap size was varied from 0 to 3 times the window size (0, 200, 400, 600) and the best value was chosen according to the criteria as previously described [36]. Distribution of peak regions relative to gene regions was analyzed by CEAS [37]. Top 10% of enriched regions for each ChIP-seq library were selected to identify the overrepresented features using EpiGRAPH (http://epigraph.mpi-inf.mpg.de/WebGRAPH/) [38]. 2214 H1d/H1c common peaks, 1939 H1d unique peaks, 433 H1c unique peaks, 1891 H3K9me3 peaks, 4778 H3K27me3 peaks, and 3446 H3K4me3 peaks were analyzed by EpiGRAPH.

### Determination of nucleosome repeat length (NRL)

ESC nuclei were extracted and MNase digestion was performed as described previously [26]. Briefly, 2.5×10^6^ nuclei were resuspended in 200 µl of MNase digestion buffer (0.32 M sucrose, 50 mM Tris-HCl pH 7.5, 4 mM MgCl_2_, 1 mM CaCl_2_, 0.1 mM PMSF) and digested at 37°C with 20 units of micrococcal nuclease (MNase) (Worthington) for time course analysis or 2 units of MNase (Worthington) for 5 min in analysis shown in Figure S10A. Nuclei were lysed and DNA was subsequently purified and analyzed by electrophoresis. Southern blotting was performed using major or minor satellite specific probes as described previously [26]. The NRL at each time point was calculated using the regression line generated with size (bp) of polynucleosomes [7], [26], and the values at time “0” were extrapolated as described previously [72].

### Fluorescence in situ hybridization (FISH)

FISH was performed as described previously [85]. The major satellite probe was biotin-labeled, denatured and hybridized to the slides overnight. The nuclei were incubated with FITC-Avidin for 1 hour, and counterstained with DAPI. Signals were detected with an Olympus Epifluorescence Microscope (Olympus, Inc.) equipped with an Olympus QCLR3 cooled digital camera. The experiments were repeated three times, and the number of chromocenters for each cell line was counted by three researchers as blind tests. Statistical analysis was performed using a Mann-Whitney U nonparametric test. Areas of chromocenters were quantitated using AxioVision software V4.8.2.0 and presented as pixel^2^. The conversion factor of pixel/micron was 18.7 pixels per micrometer.

### Quantitative reverse transcription PCR (qRT–PCR)

1 µg of total RNA extracted from ESCs was treated with RNase free DNaseI (Sigma-Aldrich) and reverse transcribed using a SuperScript first-strand cDNA synthesis kit with random hexamers (Life Technologies). Triplicate PCR reactions using the iQ SYBR Green Supermix (Bio-Rad) were analyzed in a MyIQ Real-Time PCR Detection System (Bio-Rad). All samples were typically analyzed in two independent experiments. Relative expression units were calculated by subtracting the mock reverse-transcribed signals (RT−) from reverse transcribed signals (RT+) and normalizing the adjusted values with signals of the housekeeping gene *GAPDH*. The qRT-PCR primers for repetitive sequences are the same as in qChIP, and the primers for *GAPDH* are as described previously [53].

### Bisulfite treatment of DNA and sequencing analysis

1 µg of DNA extracted from ESCs was treated with the CpGenome DNA modification Kit (Millipore) according to the manufacturer's manual. 20 ng of treated DNA was used in each PCR reaction as previously described [26]. The primers used to generate PCR products from the bisulfite-converted DNA are specific for the converted DNA sequence of the analyzed regions. The PCR products were subsequently cloned using the TOPO TA Cloning kit (Life Technologies), and colonies containing the converted DNA inserts were picked. DNA inserts were sequenced and analyzed with BiQ Analyzer [86]. Primers for major and minor satellites were as previously described [87].

## Supporting Information

Figure S1 Generation of H1d^FLAG/FLAG^ mice and H1c^Myc^ ESCs. (A) Generation and analysis of H1d^FLAG/FLAG^ mice. i) Genotyping analysis of H1d^FLAG/FLAG^ mice. The positions of WT and H1d^FLAG^ PCR bands are indicated by arrows. ii) Reverse phase HPLC and mass spectrometry analysis of extracted histones from spleens of 1-year-old wildtype (WT, left), H1c^−/−^H1d^+/+^H1e^−/−^ (ce^KO^, middle), and H1c^−/−^H1d^FLAG/FLAG^H1e^−/−^ mice (ce^KO^/H1d^FLAG/FLAG^, right). The insets are profiles generated by ESI-TOF mass spectrometry analysis of H1d/e fraction eluted from HPLC. iii) Silver staining (top) and immunoblotting (bottom) assays of individual H1 variants eluted from HPLC in (ii). 1: WT, 2: ce^KO^, 3: ce^KO^/H1d^FLAG/FLAG^. iv) H1/nucleosome ratio of histone extracts from mouse spleen. Values were calculated from HPLC analysis as shown in (ii). (B) Generation of H1c^Myc^ knock-in ESCs. i) Schematic representation of the H1c^Myc^ targeting vector and homologous recombination which results in insertion of the Myc tag at N-terminus of the coding sequence of the endogenous H1c gene. ii) Strategy of constructing H1c^Myc^ knock-in ESCs and *cis vs. trans* configurations of the homologous recombination events. iii) Reverse phase HPLC analysis of total histone extracts from H1c^Myc^ cells. iv) Coomassie stain (top) and immunoblotting (bottom) assay of the H1c and H1d/e peaks eluted from HPLC of histone extracts from ce^het^ cells and H1c^Myc^ cells.
(TIF)

Figure S2 Distribution patterns of H1 variants and histone marks at genes. (A) Examples of binding signals of H1d, H1c, and histone marks at TSSs. (B) Occupancy of H1 variants and histone marks at 4 *Hox* clusters. (C) Metagene profiling analysis of H3K9me3 (left), H3K27me3 (middle) and H3K4me3 (right) around TSS in relation to levels of themselves.
(TIF)

Figure S3 Progressively elevated levels of H1 variants with increasing distance from TSS. Signal values of 100 bp windows at TSS and indicated flanking regions of genes with lowest H1 values (20% of all genes) were plotted. Distal data points situated in the vicinity of other TSSs were removed from calculation. P<10^−50^ for all comparisons (with TSS) with paired t-test. The line in the box indicates the median, while the bottom and top of the boxes are the 25^th^ and 75^th^ percentiles, respectively. The red line represents the median signals at +/−10 Mb distal to TSS.
(TIF)

Figure S4 Annotation and distribution analysis of H1d and H1c enriched regions. (A) Examples of H1d and H1c enriched regions. (B) Pie diagram of distributions of H1d, H1c, H3K9me3, H3K27me3, and H3K4me3 enriched regions at genes, proximal regulatory regions, and distal intergenic regions.
(TIF)

Figure S5 Occupancy correlation and overrepresentation analysis of H1 variants and histone marks. (A) Correlation coefficients of H1d *vs.* H1c, each H1 variant (H1d or H1c) *vs.* GC percentage and histone marks on individual chromosomes. Pearson's correlation was used to perform the analysis. P<10^−100^ for all correlation coefficients except for those labeled with “*”. *: P>0.001. (B) EpiGRAPH overrepresentation analyses of comparisons of H1d (or H1c) uniquely enriched regions *vs.* histone marks enriched regions as described in methods. H1d unique regions (left panels) or (H1c unique regions (right panels)) *vs.* H3K9me3 regions (i), *vs.* H3K27me3 regions (ii), *vs.* H3K4me3 regions (iii). Overrepresented repetitive elements are shown in the bottom half of each box. *: no significantly overrepresented features. All P values remained significant after multiple testing corrections with the FDR method and the more conservative Bonferroni method.
(TIF)

Figure S6 qChIP analysis of H1d, H1c and histone marks at selected repetitive elements. Relative enrichment was calculated by normalizing the signals of ChIP over that of IgG. Data are presented as mean ± S.D.
(TIF)

Figure S7 Distribution of H1d, H1c, and histone marks on additional repetitive sequences. Fold enrichment of percent mapped repeats of H1d, H1c, H3K9me3, H3K27me3, and H3K4me3 ChIP-seq libraries over that of corresponding input-seq library. 14 most abundant repetitive sequences within the “other” repetitive group shown in Figure 4B are presented. P values calculated with Fisher's exact test comparing ChIP-seq with input-seq libraries are less than 1.3×10^−7^ for all repeat classes shown except those marked with “*”. *: P>0.01. Error bars represent the differences between replicates. Data are presented as average ± S.E.M.
(TIF)

Figure S8 Significant enrichment of H1 variants at major satellites. Box plots of the signals of three H1 variants and histone marks at major satellite repeats (left panel); TSS, 10 Mb distal to TSS, LINE L1, and IAP LTR repeats (right panel). Y axis: input subtracted, normalized signal values as tag counts per 100 bp window per 10 million mappable reads. The line in the box indicates the median, while the bottom and top of the boxes are the 25^th^ and 75^th^ percentiles, respectively. P<4×10^−6^ for all comparisons with H3K9me3 within each category by unpaired t-tests.
(TIF)

Figure S9 Analysis of H1d-*trans* ESC line. (A) Reverse phase HPLC of total histone extracts from H1d-*trans* cells. (B) Ratios of each H1 variant (and total H1) to nucleosome of H1d-*trans* cells calculated from data shown in (A). (C) Western blotting indicating similar amount of FLAG-H1d in H1d^FLAG^ and H1d-*trans* cells. (D) Fold enrichment of percent mapped repeats of H1d in H1d^FLAG^ and H1d-*trans* ESCs. P values comparing ChIP-seq with input-seq libraries are less than 9.3×10^−14^ for all repeat classes shown. (E) qChIP analysis of H1d occupancy at indicated repetitive elements in H1d-*trans* cells. Relative enrichment was calculated by normalizing the signals of ChIP over that of IgG.
(TIF)

Figure S10 H1 depletion leads to reduced NRLs and H1 occupancy at major and minor satellites. (A) Southern blotting analysis of partially digested nuclei using a major satellite probe. The tetra-nucleosome bands are indicated by asterisks. (B–C) Elevated NRLs at major satellites compared with bulk chromatin and minor satellites in H1 TKO ESCs. Data from EB-stained gel image (B, left) and corresponding Southern blots (B, middle and right) are plotted in (C). The positions of di-nucleosome with a 10-minute MNase digestion are marked by asterisks in (B). The dashed line in (B) indicates the di-nuleosome position in major satellites, which is higher than that of bulk chromatin and minor satellites. NRLs in (C) were calculated by extrapolating the corresponding curves to time 0 as described [72]. (D) qChIP analysis of H1 occupancy at major satellites, minor satellites, and *HPRT* gene in WT and H1 TKO ESCs. ChIP signals over IgG levels are presented as mean ± S.D. *: P<0.05; **: P<0.01; ***: P<0.001.
(TIF)

Figure S11 Chromocenter area in WT, H1 TKO and RES ESCs. Chromocenters of 80 nuclei from each cell line were analyzed. The line in the box indicates the median, while the bottom and top of the boxes are the 25^th^ and 75^th^ percentiles, respectively. ****: P<0.000001.
(TIF)

Figure S12 Generation of H1^0^ over-expressing (fH1^0^) ESCs. (A) Representative Western blots of H1^0^ over-expressing cell clones. WT ESCs were transfected with vector expressing FLAG-H1^0^, and stable ESC clones were picked and screened using an anti-FLAG antibody. Immunoblotting with anti-β-ACTIN antibody indicates equal loading of whole cell lysates. An H1^0^ overexpressing clone with significant levels of FLAG-H1^0^ is indicated with an asterisk. (B) RP-HPLC Profile of fH1^0^ ESCs. (C) Ratio of individual H1 variant (and total H1) to nucleosome of fH1^0^ ESCs calculated from HPLC profile shown in (B). (D) Western blots indicating similar levels of H1^0^ in H1 TKO cells and the FLAG-H1^0^ in fH1^0^ ESCs. H3 blots indicates equal loading of chromatin lysates.
(TIF)

Figure S13 H1^0^ is depleted from active promoters. (A) Examples of H1^0^ distribution at TSSs. (B–F) Metagene analysis of H1^0^ levels over a 10 Kb window centered on TSSs partitioned according to the levels of expression (B), H3K9me3 (C), H3K4me3 (D), H3K27me3 (E), and the presence or absence of H3K4me3 and H3K27me3 (F).
(TIF)

Figure S14 H1^0^ is enriched at satellite sequences. (A) A typical peak region of H1^0^ at major satellites. (B) Fold enrichment of percent mappable repeats from the H1^0^ ChIP-seq library over that of input-seq library on total repeats (left) and 20 most abundant repetitive sequences (right). P values calculated with Fisher's exact test comparing ChIP-seq with input-seq libraries are less than 1.8×10^−21^ for all repeats shown except those marked with “*”. *: P>0.01. (C) qChIP analysis of H1^0^ occupancy at selected repetitive sequences. Relative enrichment was calculated by normalizing the signals of ChIP over that of IgG.
(TIF)

Figure S15 FISH analyses of chromocenters in H1d^FLAG^, H1c^Myc^, and fH1^0^ ESCs. (A) Typical FISH images of indicated cells hybridized with a major satellite probe are shown in left panels. DNA was counterstained with DAPI (middle), and merged images are shown in right panels. Scale bar: 10 µm. (B) Box plots of the numbers of chromocenters in indicated ESCs. The line in the box indicates the median, while the bottom and top of the boxes are the 25^th^ and 75^th^ percentiles, respectively.
(TIF)

Figure S16 Expression analysis of major satellites in fH1^0^, H1d^FLAG^, and H1c^Myc^ ESCs. Data are shown as mean ± S.D. a: P<0.05 in comparison with WT; b: P<0.05 in comparison with TKO.
(TIF)

Table S1 List of read length, counts, and total mappable reads (to mm9) of the libraries.
(DOC)
