# Supplementary figures and images for: High-Resolution Mapping of H1 Linker Histone Variants in Embryonic Stem Cells
Source: PLoS Genet. 2013 Apr 25;9(4):e1003417. doi: 10.1371/journal.pgen.1003417 (PMC3636266; doi:10.1371/journal.pgen.1003417)

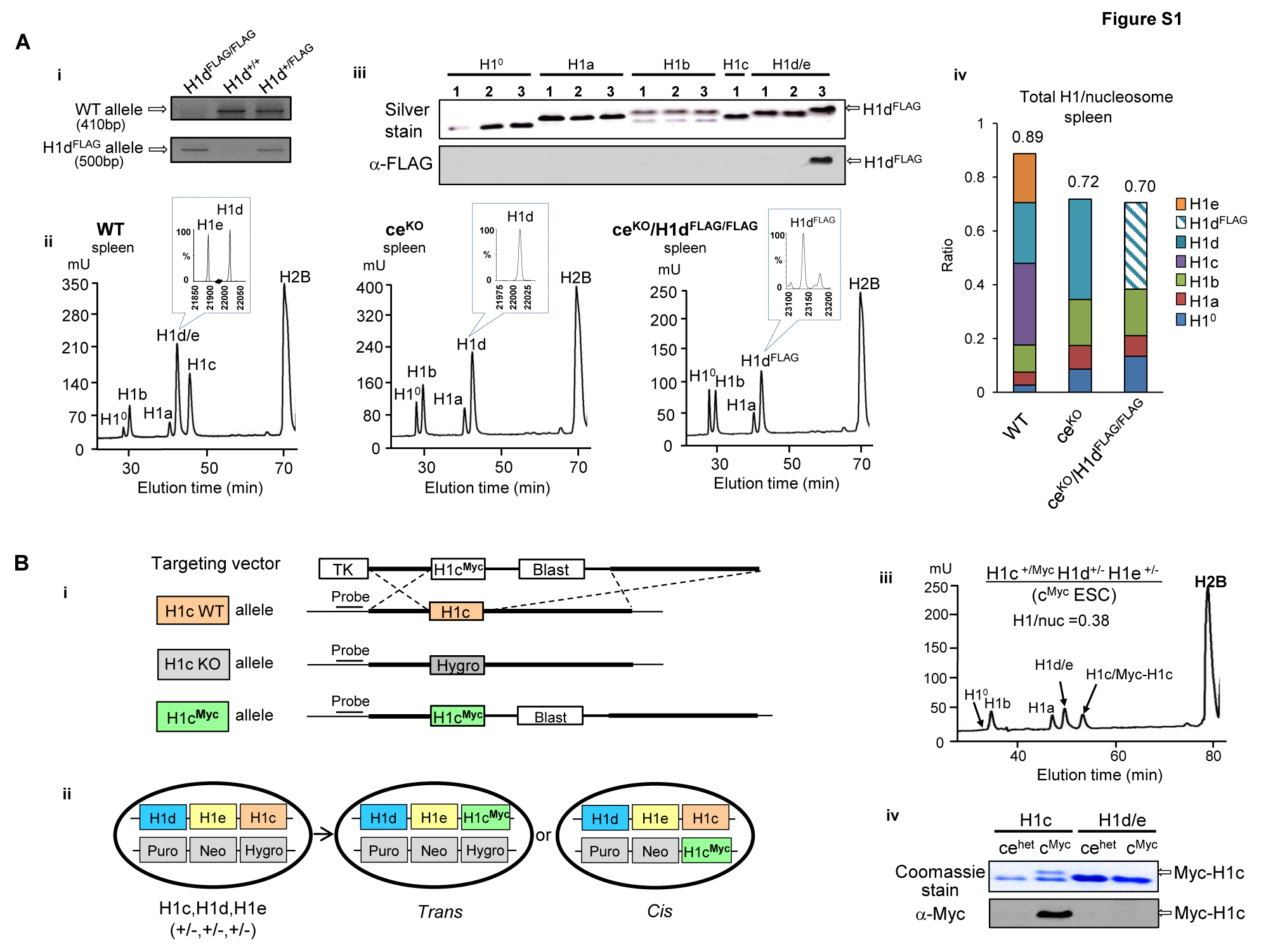

Supplement: Figure S1 — Generation of H1dFLAG/FLAG mice and H1cMyc ESCs. (A) Generation and analysis of H1dFLAG/FLAG mice. i) Genotyping analysis of H1dFLAG/FLAG mice. The positions of WT and H1dFLAG PCR bands are indicated by arrows. ii) Reverse phase HPLC and mass spectrometry analysis of extracted histones from spleens of 1-year-old wildtype (WT, left), H1c−/−H1d+/+H1e−/− (ceKO, middle), and H1c−/−H1dFLAG/FLAGH1e−/− mice (ceKO/H1dFLAG/FLAG, right). The insets are profiles generated by ESI-TOF mass spectrometry analysis of H1d/e fraction eluted from HPLC. iii) Silver staining (top) and immunoblotting (bottom) assays of individual H1 variants eluted from HPLC in (ii). 1: WT, 2: ceKO, 3: ceKO/H1dFLAG/FLAG. iv) H1/nucleosome ratio of histone extracts from mouse spleen. Values were calculated from HPLC analysis as shown in (ii). (B) Generation of H1cMyc knock-in ESCs. i) Schematic representation of the H1cMyc targeting vector and homologous recombination which results in insertion of the Myc tag at N-terminus of the coding sequence of the endogenous H1c gene. ii) Strategy of constructing H1cMyc knock-in ESCs and cis vs. trans configurations of the homologous recombination events. iii) Reverse phase HPLC analysis of total histone extracts from H1cMyc cells. iv) Coomassie stain (top) and immunoblotting (bottom) assay of the H1c and H1d/e peaks eluted from HPLC of histone extracts from cehet cells and H1cMyc cells. (TIF) [file pgen.1003417.s001.tif]

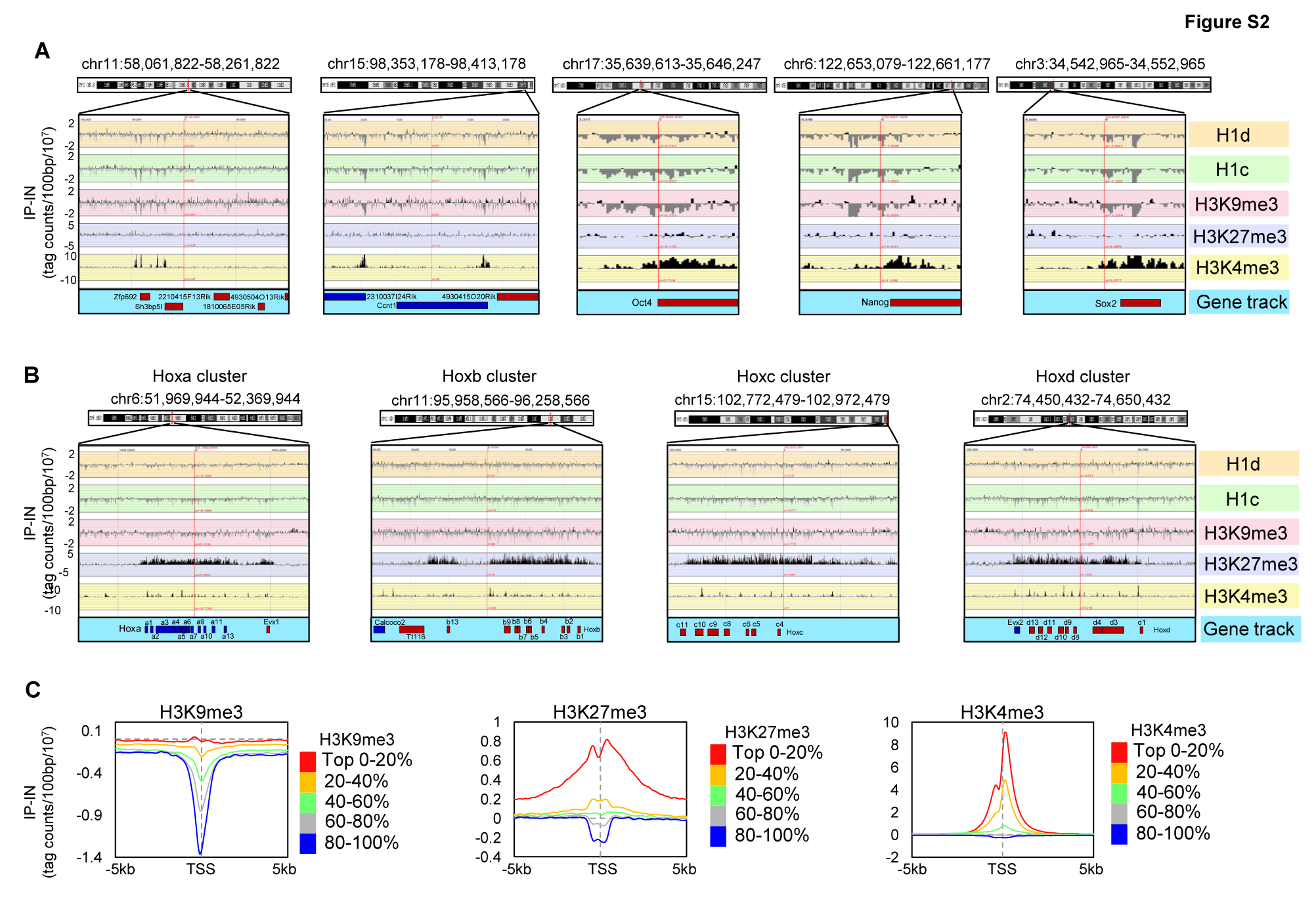

Supplement: Figure S2 — Distribution patterns of H1 variants and histone marks at genes. (A) Examples of binding signals of H1d, H1c, and histone marks at TSSs. (B) Occupancy of H1 variants and histone marks at 4 Hox clusters. (C) Metagene profiling analysis of H3K9me3 (left), H3K27me3 (middle) and H3K4me3 (right) around TSS in relation to levels of themselves. (TIF) [file pgen.1003417.s002.tif]

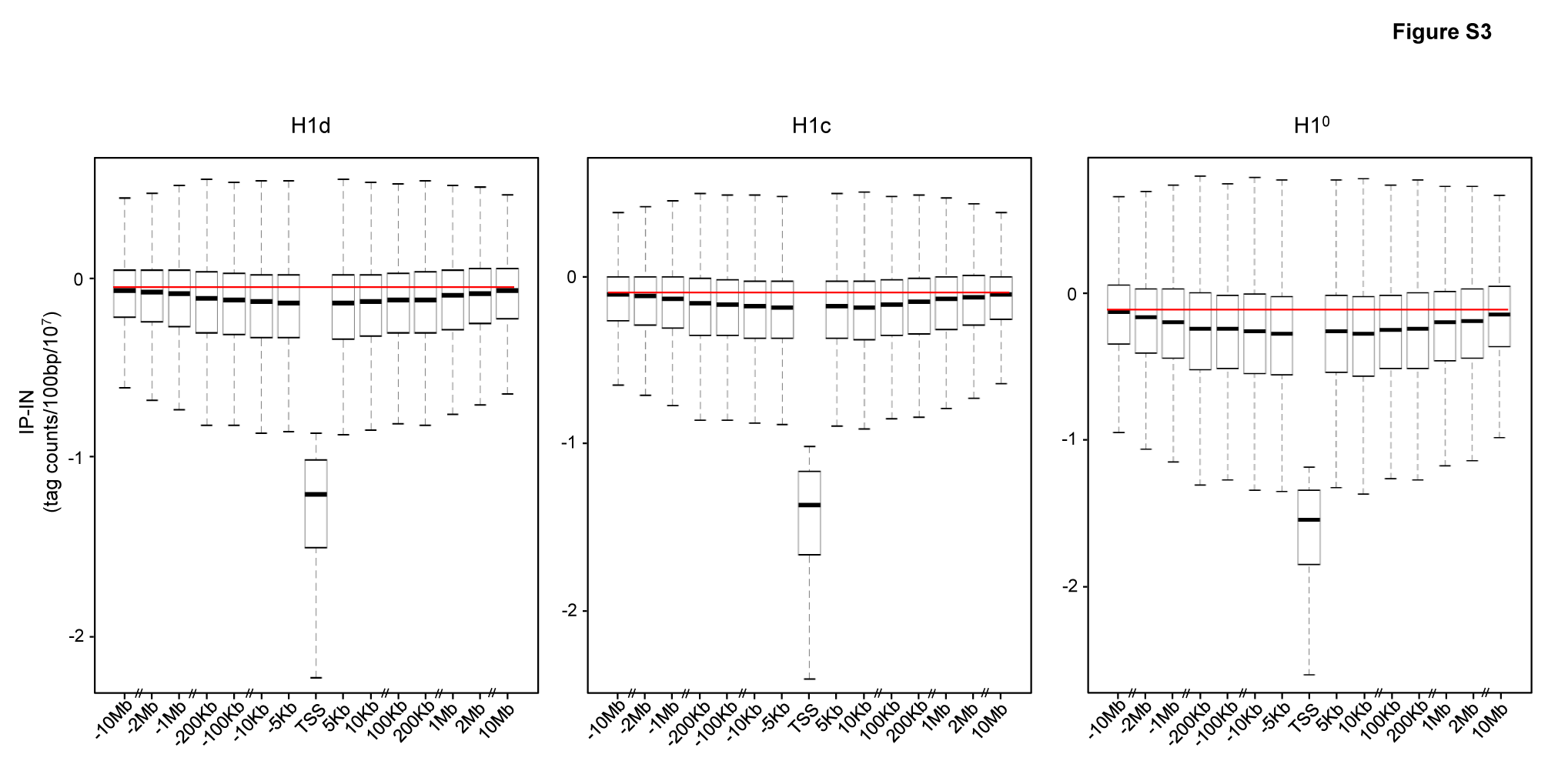

Supplement: Figure S3 — Progressively elevated levels of H1 variants with increasing distance from TSS. Signal values of 100 bp windows at TSS and indicated flanking regions of genes with lowest H1 values (20% of all genes) were plotted. Distal data points situated in the vicinity of other TSSs were removed from calculation. P<10−50 for all comparisons (with TSS) with paired t-test. The line in the box indicates the median, while the bottom and top of the boxes are the 25th and 75th percentiles, respectively. The red line represents the median signals at +/−10 Mb distal to TSS. (TIF) [file pgen.1003417.s003.tif]

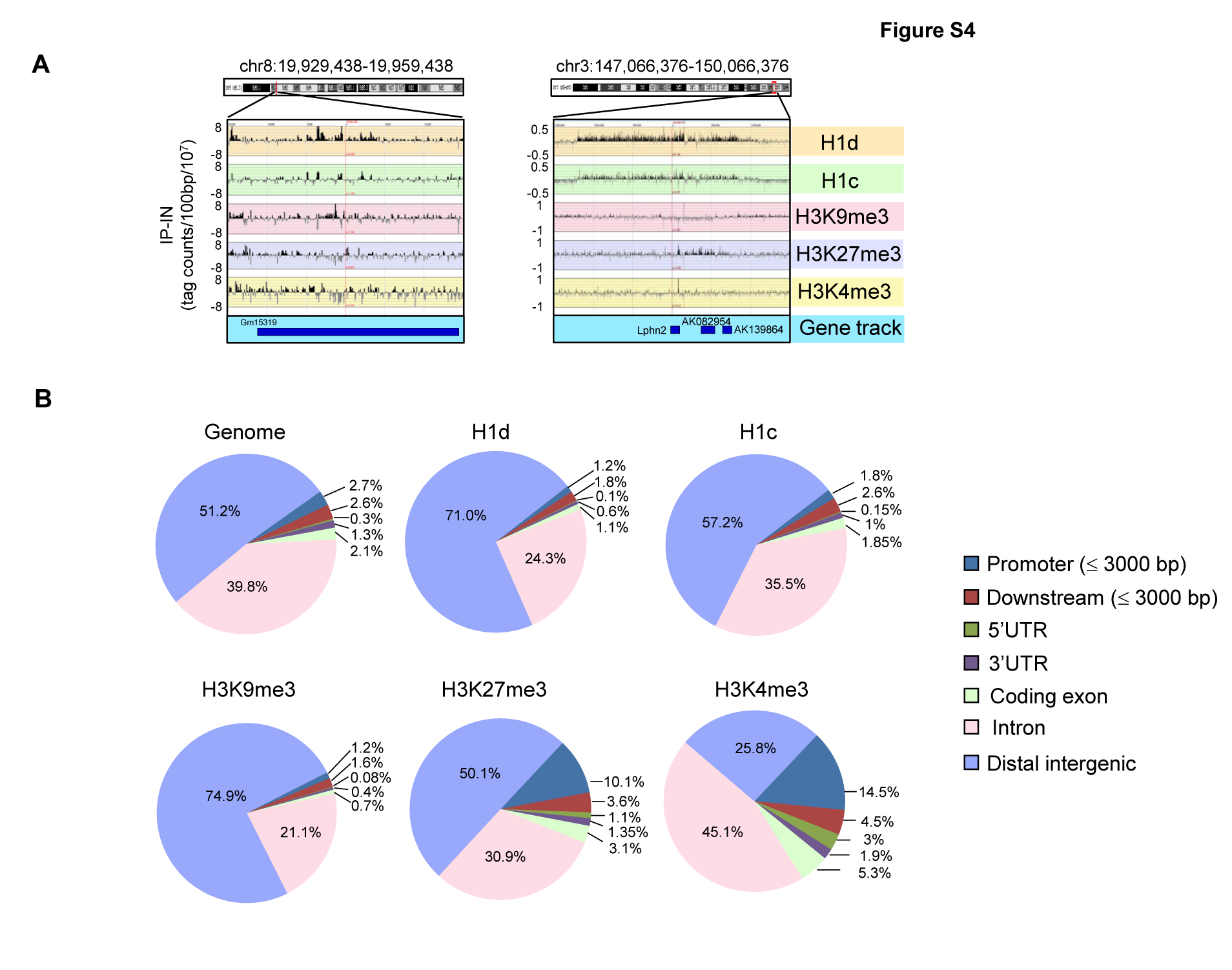

Supplement: Figure S4 — Annotation and distribution analysis of H1d and H1c enriched regions. (A) Examples of H1d and H1c enriched regions. (B) Pie diagram of distributions of H1d, H1c, H3K9me3, H3K27me3, and H3K4me3 enriched regions at genes, proximal regulatory regions, and distal intergenic regions. (TIF) [file pgen.1003417.s004.tif]

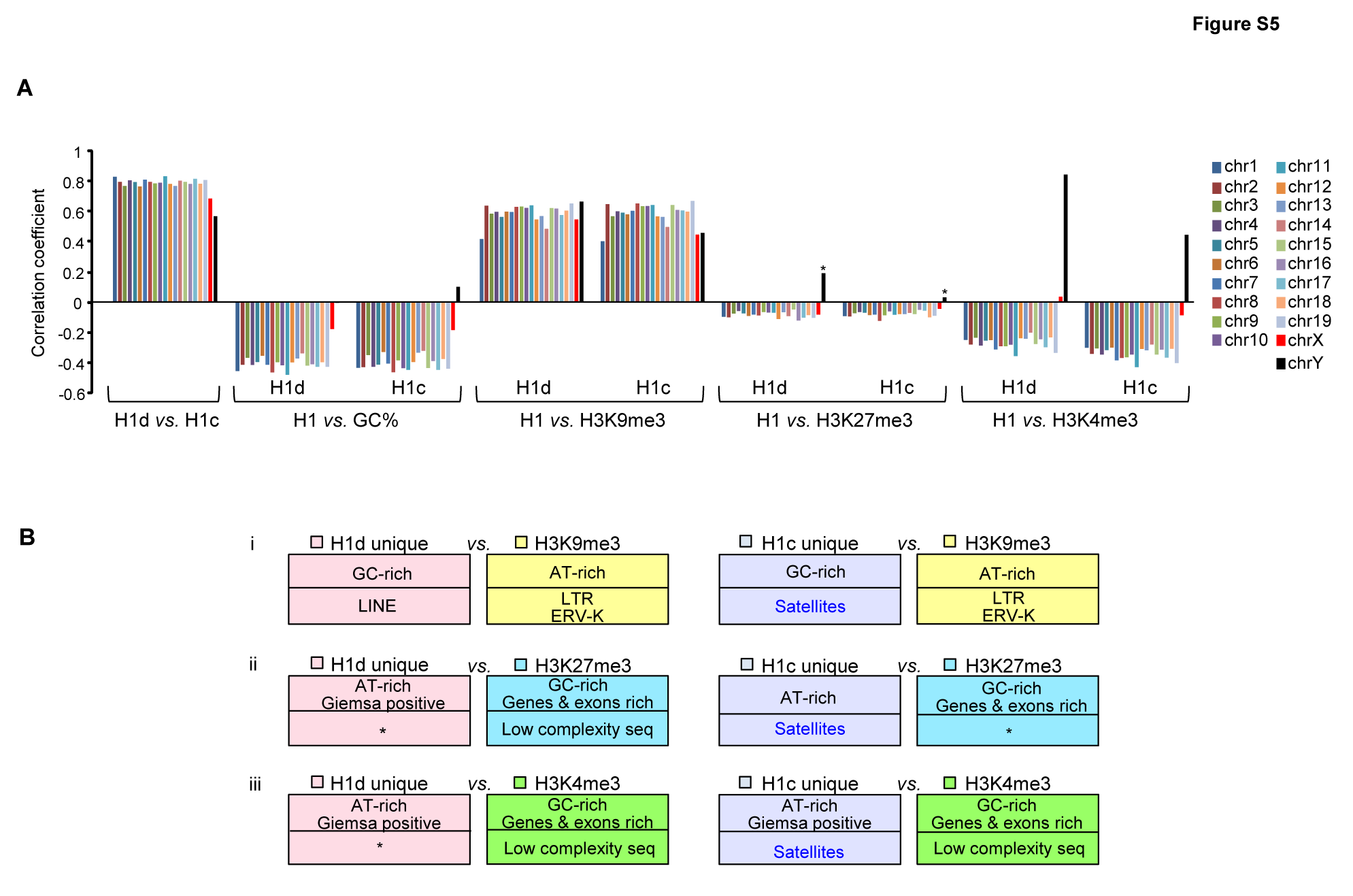

Supplement: Figure S5 — Occupancy correlation and overrepresentation analysis of H1 variants and histone marks. (A) Correlation coefficients of H1d vs. H1c, each H1 variant (H1d or H1c) vs. GC percentage and histone marks on individual chromosomes. Pearson's correlation was used to perform the analysis. P<10−100 for all correlation coefficients except for those labeled with “*”. *: P>0.001. (B) EpiGRAPH overrepresentation analyses of comparisons of H1d (or H1c) uniquely enriched regions vs. histone marks enriched regions as described in methods. H1d unique regions (left panels) or (H1c unique regions (right panels)) vs. H3K9me3 regions (i), vs. H3K27me3 regions (ii), vs. H3K4me3 regions (iii). Overrepresented repetitive elements are shown in the bottom half of each box. *: no significantly overrepresented features. All P values remained significant after multiple testing corrections with the FDR method and the more conservative Bonferroni method. (TIF) [file pgen.1003417.s005.tif]

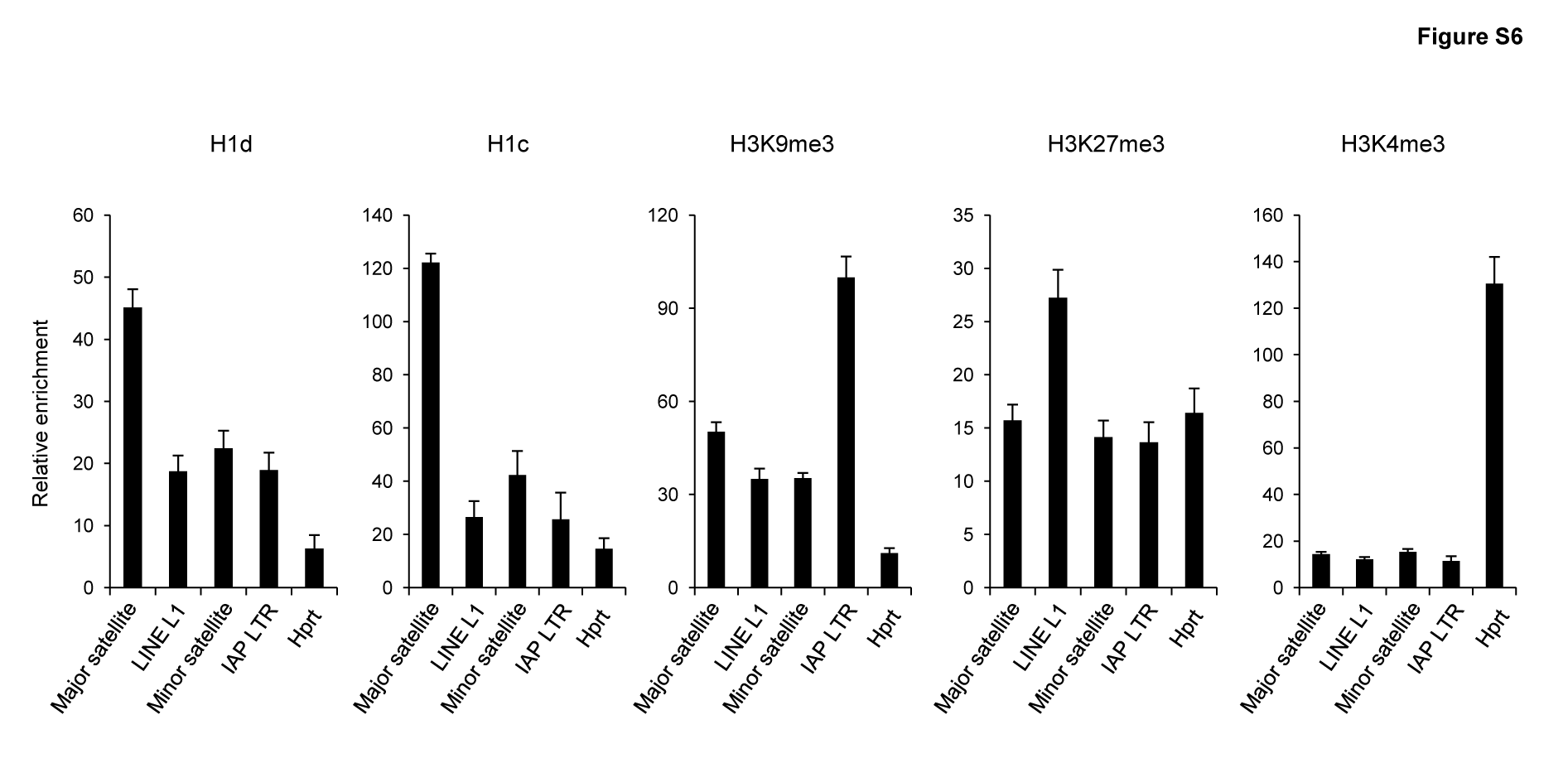

Supplement: Figure S6 — qChIP analysis of H1d, H1c and histone marks at selected repetitive elements. Relative enrichment was calculated by normalizing the signals of ChIP over that of IgG. Data are presented as mean ± S.D. (TIF) [file pgen.1003417.s006.tif]

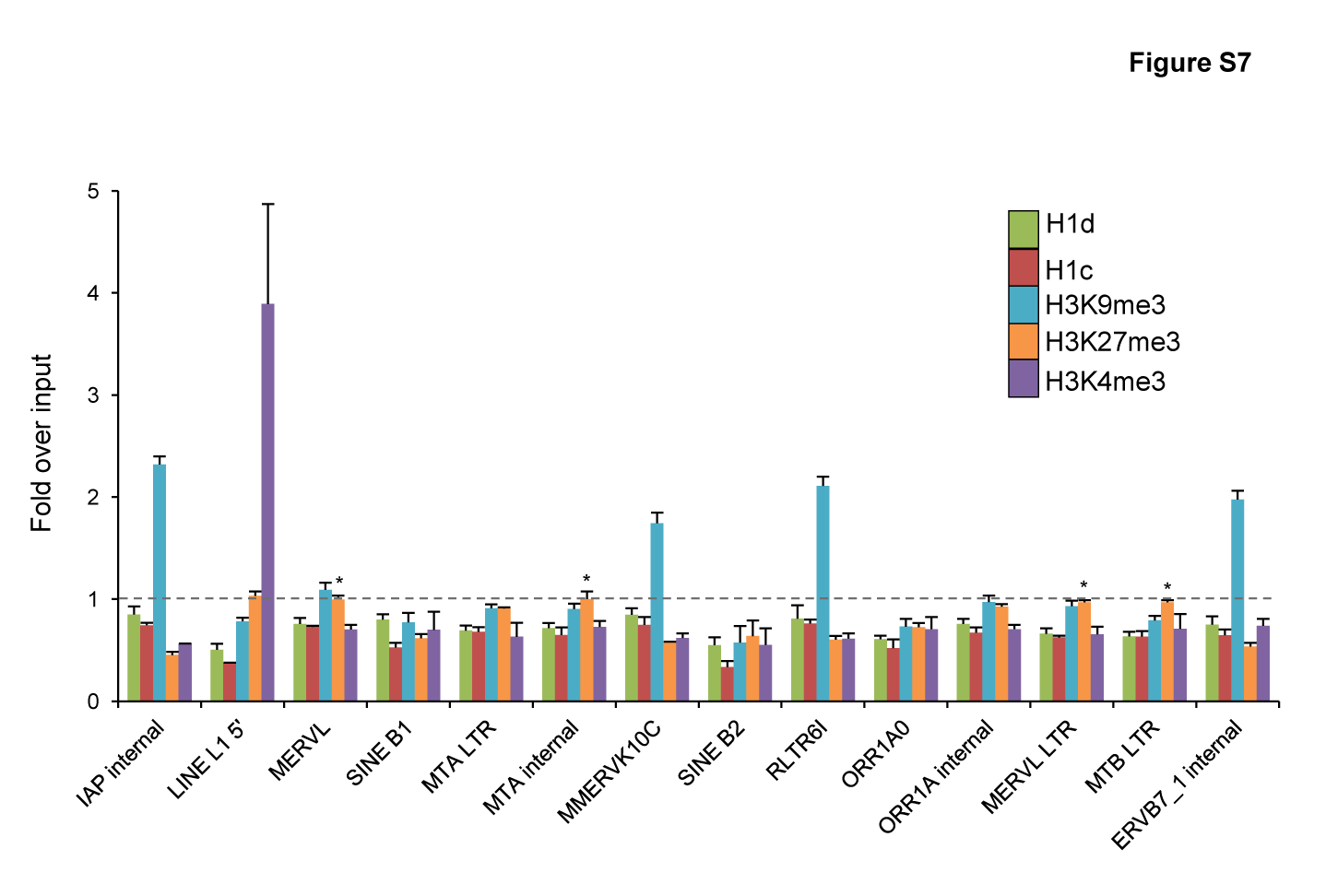

Supplement: Figure S7 — Distribution of H1d, H1c, and histone marks on additional repetitive sequences. Fold enrichment of percent mapped repeats of H1d, H1c, H3K9me3, H3K27me3, and H3K4me3 ChIP-seq libraries over that of corresponding input-seq library. 14 most abundant repetitive sequences within the “other” repetitive group shown in Figure 4B are presented. P values calculated with Fisher's exact test comparing ChIP-seq with input-seq libraries are less than 1.3×10^−7^ for all repeat classes shown except those marked with “*”. *: P>0.01. Error bars represent the differences between replicates. Data are presented as average ± S.E.M. (TIF) [file pgen.1003417.s007.tif]

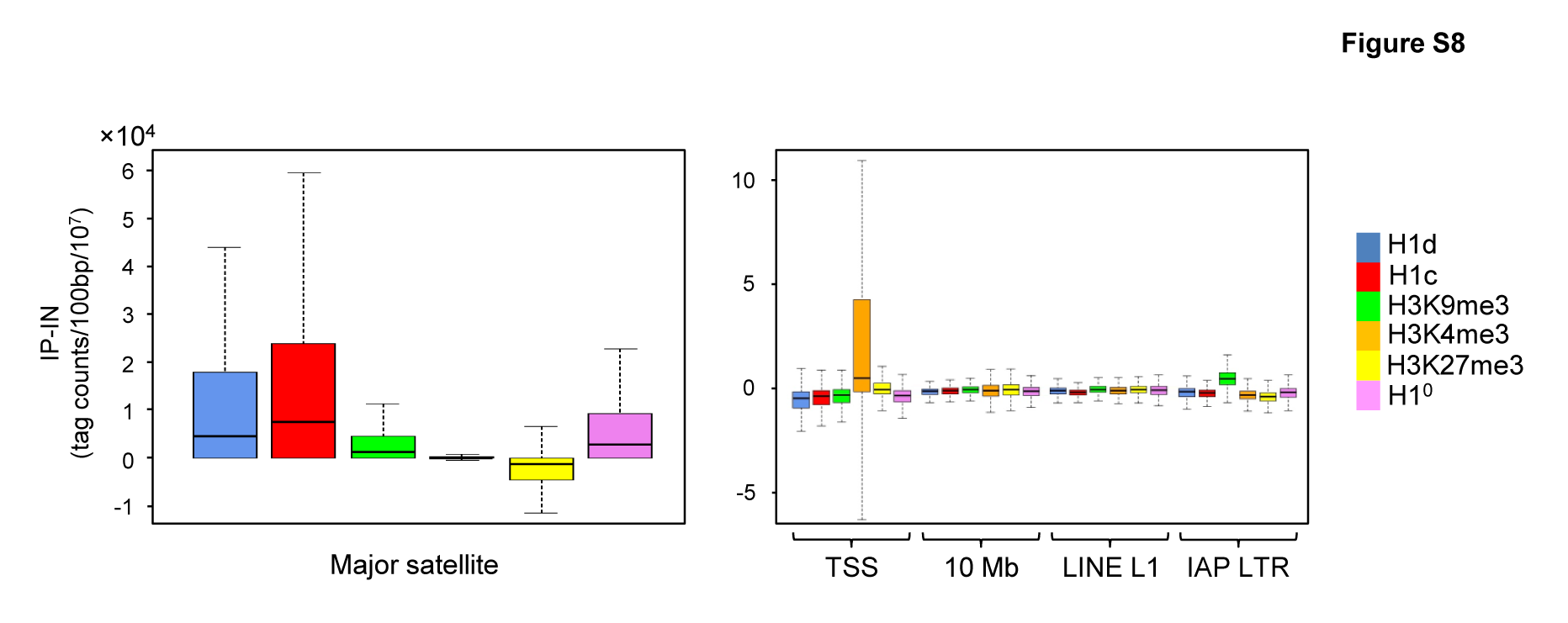

Supplement: Figure S8 — Significant enrichment of H1 variants at major satellites. Box plots of the signals of three H1 variants and histone marks at major satellite repeats (left panel); TSS, 10 Mb distal to TSS, LINE L1, and IAP LTR repeats (right panel). Y axis: input subtracted, normalized signal values as tag counts per 100 bp window per 10 million mappable reads. The line in the box indicates the median, while the bottom and top of the boxes are the 25th and 75th percentiles, respectively. P<4×10^−6^ for all comparisons with H3K9me3 within each category by unpaired t-tests. (TIF) [file pgen.1003417.s008.tif]

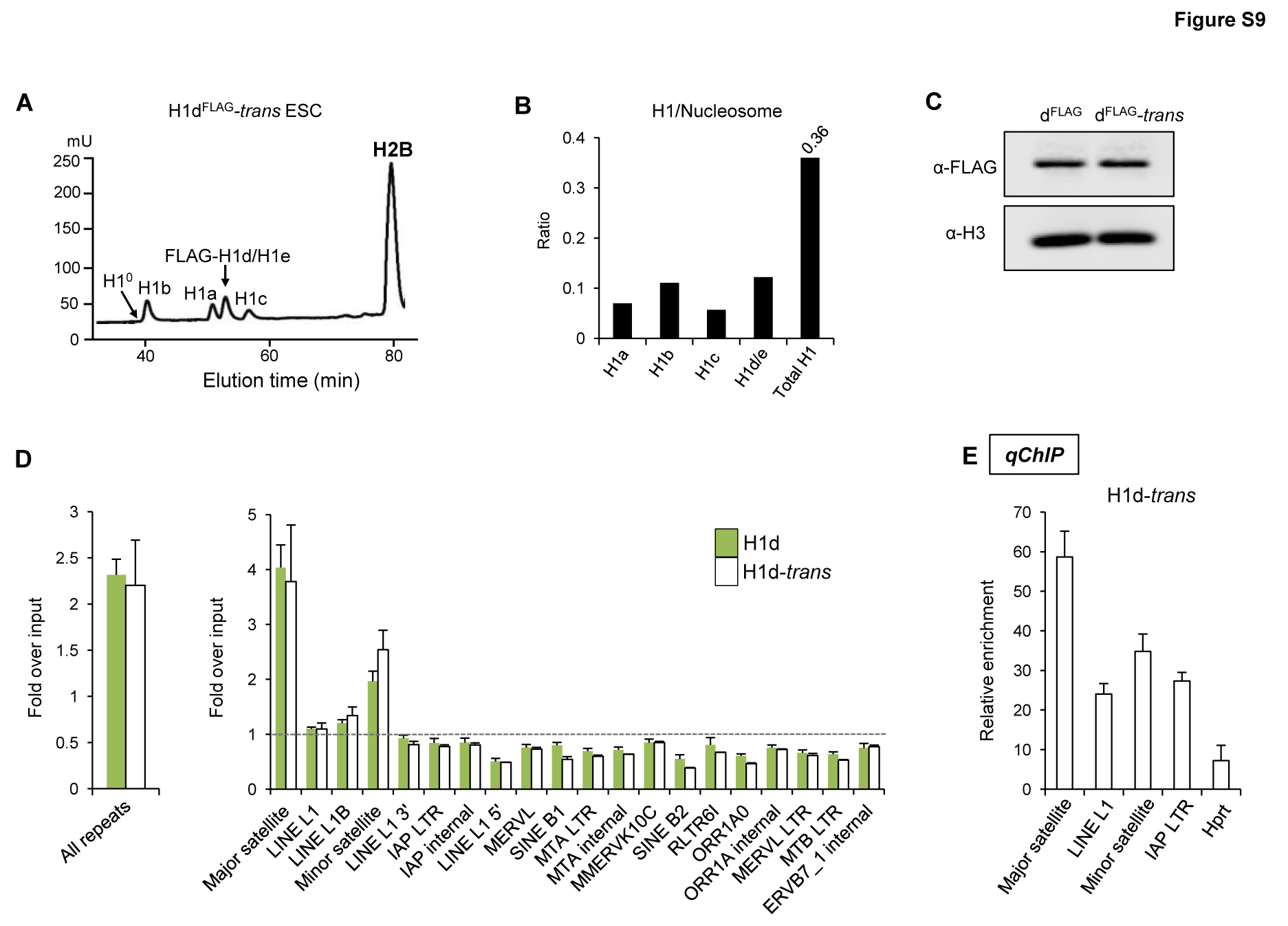

Supplement: Figure S9 — Analysis of H1d-trans ESC line. (A) Reverse phase HPLC of total histone extracts from H1d-trans cells. (B) Ratios of each H1 variant (and total H1) to nucleosome of H1d-trans cells calculated from data shown in (A). (C) Western blotting indicating similar amount of FLAG-H1d in H1dFLAG and H1d-trans cells. (D) Fold enrichment of percent mapped repeats of H1d in H1dFLAG and H1d-trans ESCs. P values comparing ChIP-seq with input-seq libraries are less than 9.3×10^−14^ for all repeat classes shown. (E) qChIP analysis of H1d occupancy at indicated repetitive elements in H1d-trans cells. Relative enrichment was calculated by normalizing the signals of ChIP over that of IgG. (TIF) [file pgen.1003417.s009.tif]

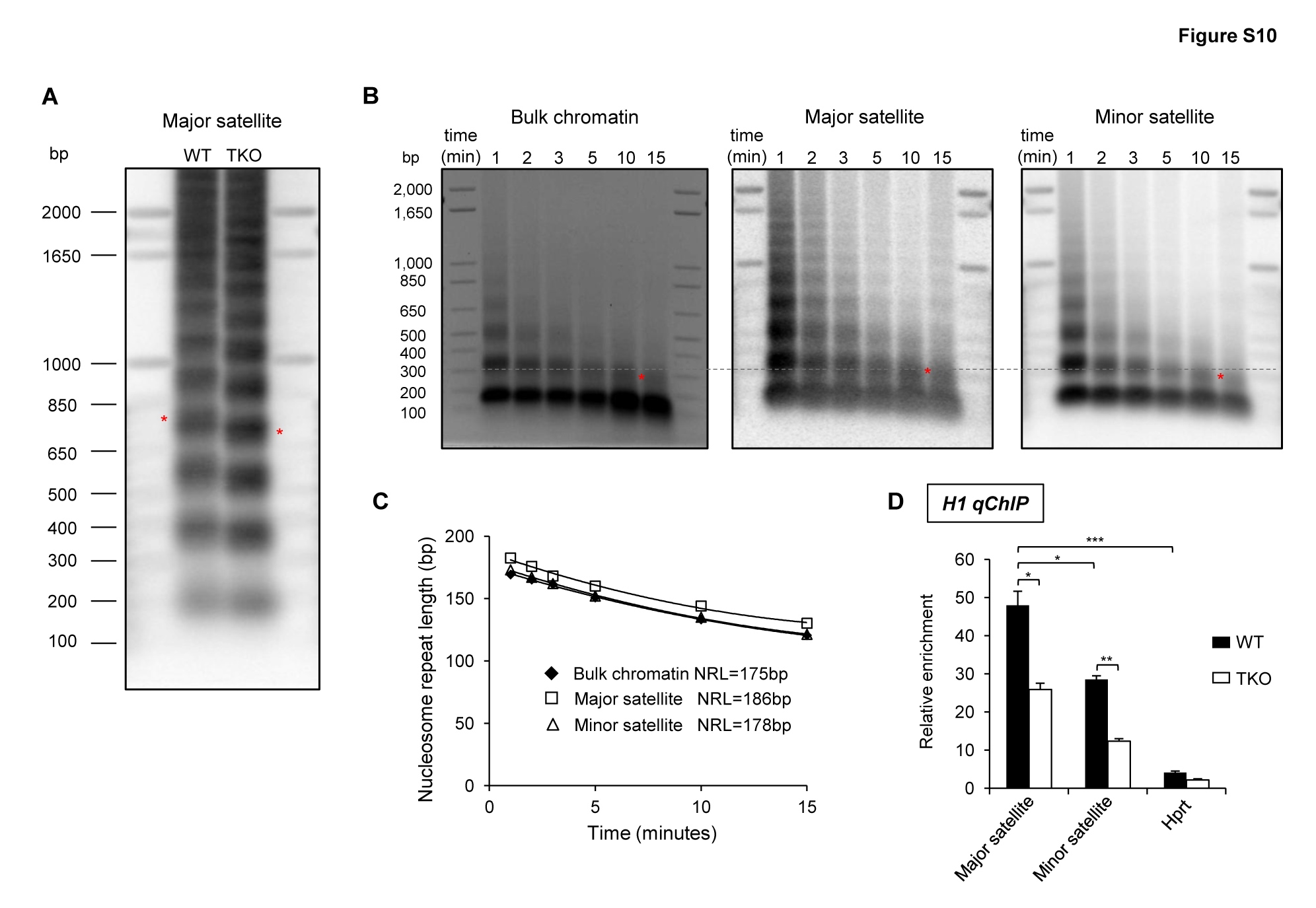

Supplement: Figure S10 — H1 depletion leads to reduced NRLs and H1 occupancy at major and minor satellites. (A) Southern blotting analysis of partially digested nuclei using a major satellite probe. The tetra-nucleosome bands are indicated by asterisks. (B–C) Elevated NRLs at major satellites compared with bulk chromatin and minor satellites in H1 TKO ESCs. Data from EB-stained gel image (B, left) and corresponding Southern blots (B, middle and right) are plotted in (C). The positions of di-nucleosome with a 10-minute MNase digestion are marked by asterisks in (B). The dashed line in (B) indicates the di-nuleosome position in major satellites, which is higher than that of bulk chromatin and minor satellites. NRLs in (C) were calculated by extrapolating the corresponding curves to time 0 as described [72]. (D) qChIP analysis of H1 occupancy at major satellites, minor satellites, and HPRT gene in WT and H1 TKO ESCs. ChIP signals over IgG levels are presented as mean ± S.D. *: P<0.05; **: P<0.01; ***: P<0.001. (TIF) [file pgen.1003417.s010.tif]

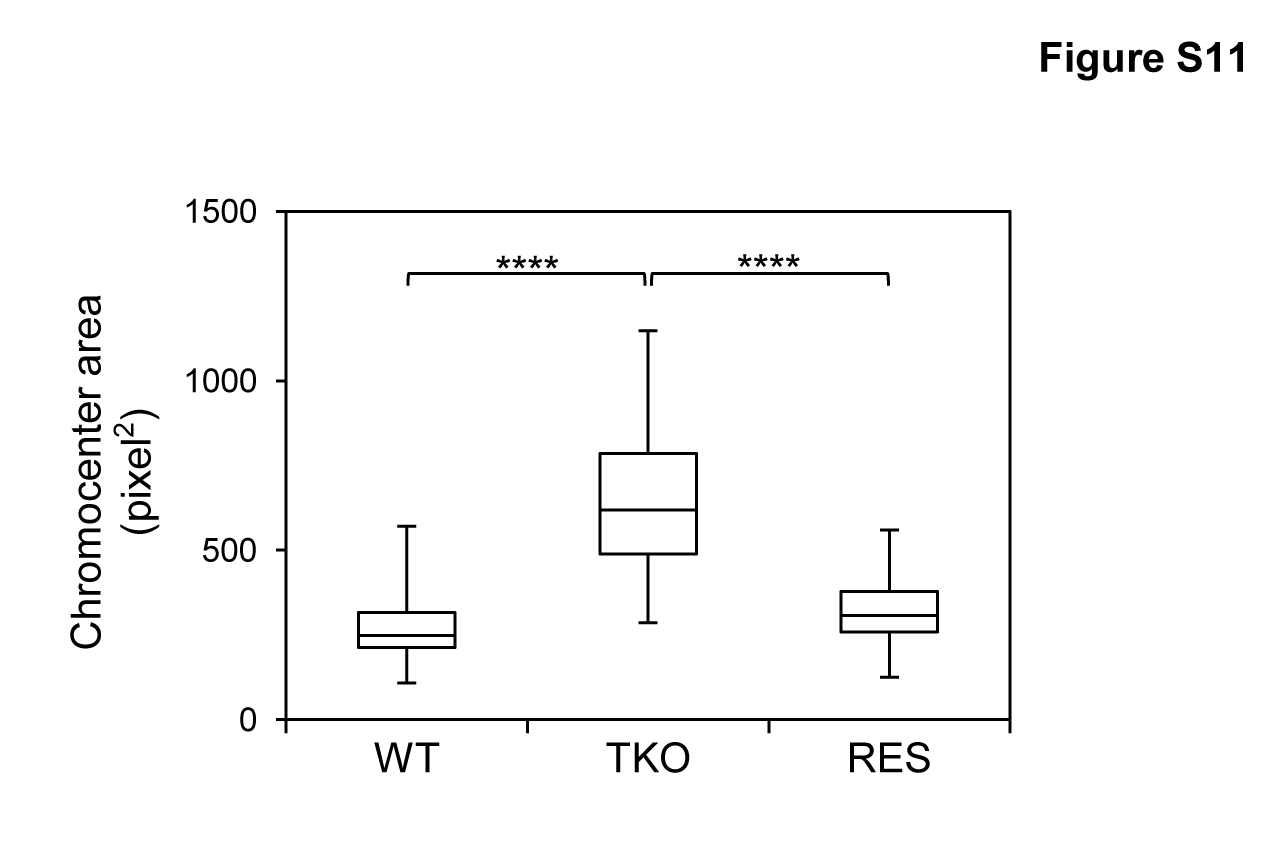

Supplement: Figure S11 — Chromocenter area in WT, H1 TKO and RES ESCs. Chromocenters of 80 nuclei from each cell line were analyzed. The line in the box indicates the median, while the bottom and top of the boxes are the 25th and 75th percentiles, respectively. ****: P<0.000001. (TIF) [file pgen.1003417.s011.tif]

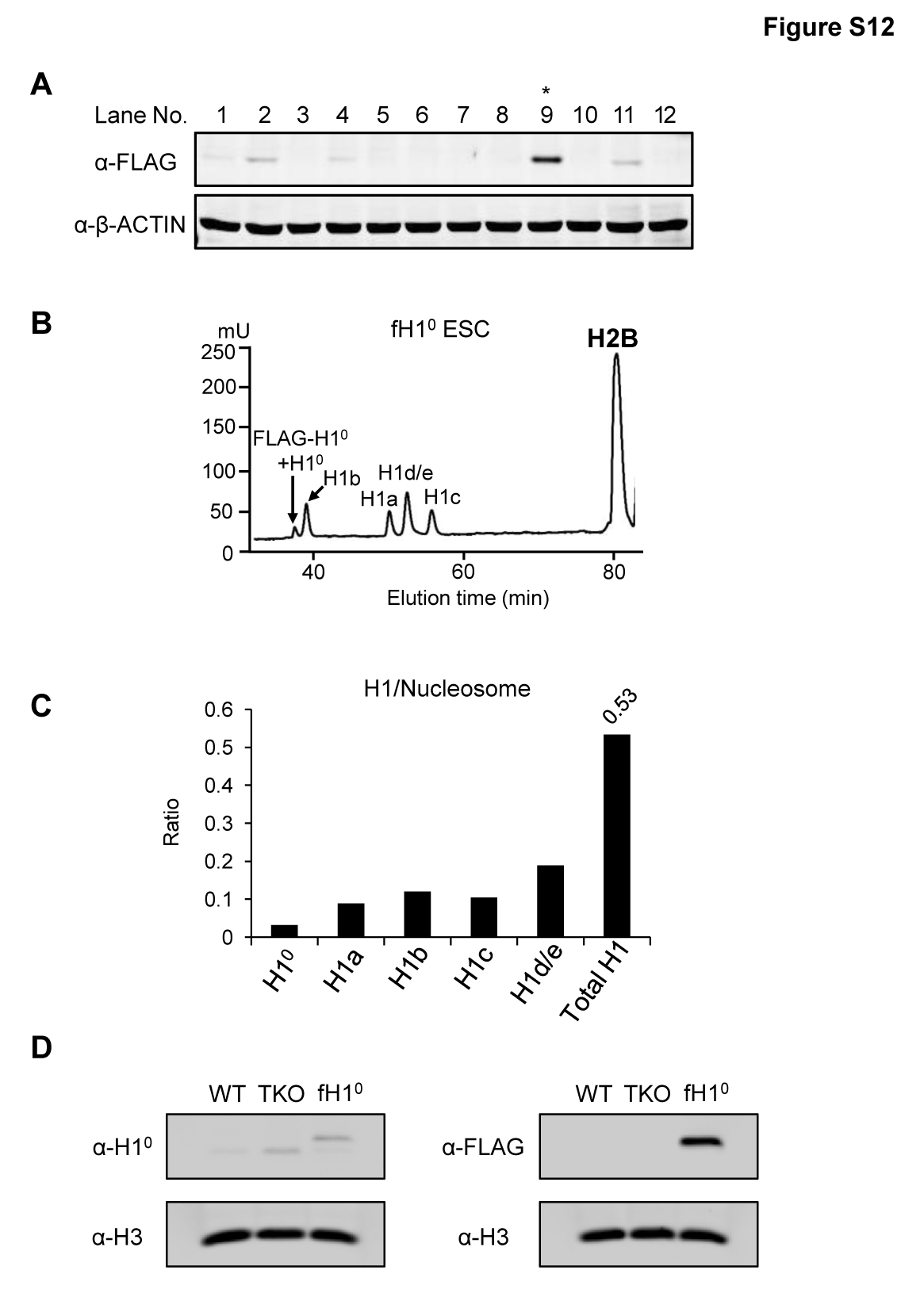

Supplement: Figure S12 — Generation of H10 over-expressing (fH10) ESCs. (A) Representative Western blots of H10 over-expressing cell clones. WT ESCs were transfected with vector expressing FLAG-H10, and stable ESC clones were picked and screened using an anti-FLAG antibody. Immunoblotting with anti-β-ACTIN antibody indicates equal loading of whole cell lysates. An H10 overexpressing clone with significant levels of FLAG-H10 is indicated with an asterisk. (B) RP-HPLC Profile of fH10 ESCs. (C) Ratio of individual H1 variant (and total H1) to nucleosome of fH10 ESCs calculated from HPLC profile shown in (B). (D) Western blots indicating similar levels of H10 in H1 TKO cells and the FLAG-H10 in fH10 ESCs. H3 blots indicates equal loading of chromatin lysates. (TIF) [file pgen.1003417.s012.tif]

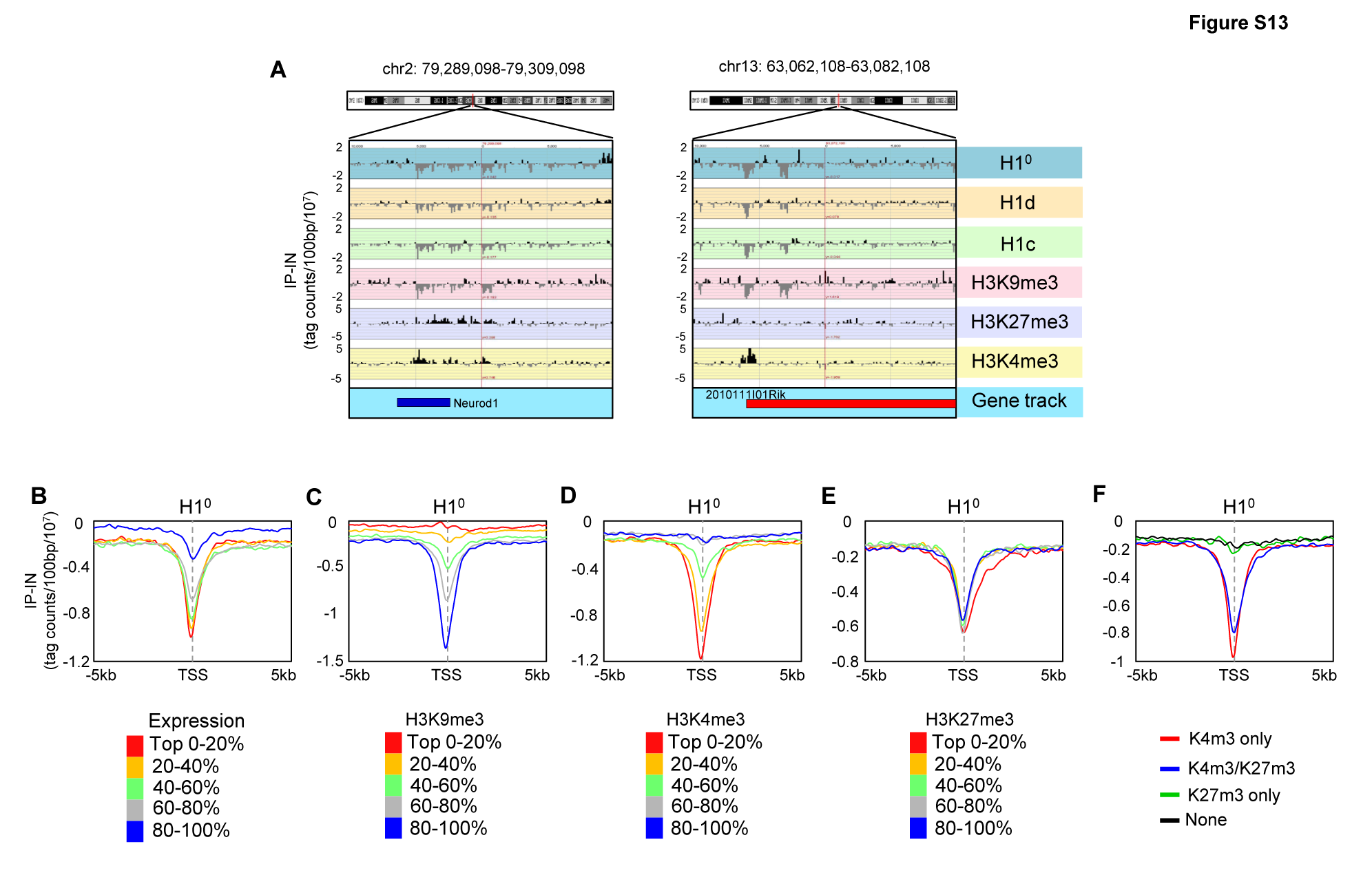

Supplement: Figure S13 — H10 is depleted from active promoters. (A) Examples of H10 distribution at TSSs. (B–F) Metagene analysis of H10 levels over a 10 Kb window centered on TSSs partitioned according to the levels of expression (B), H3K9me3 (C), H3K4me3 (D), H3K27me3 (E), and the presence or absence of H3K4me3 and H3K27me3 (F). (TIF) [file pgen.1003417.s013.tif]

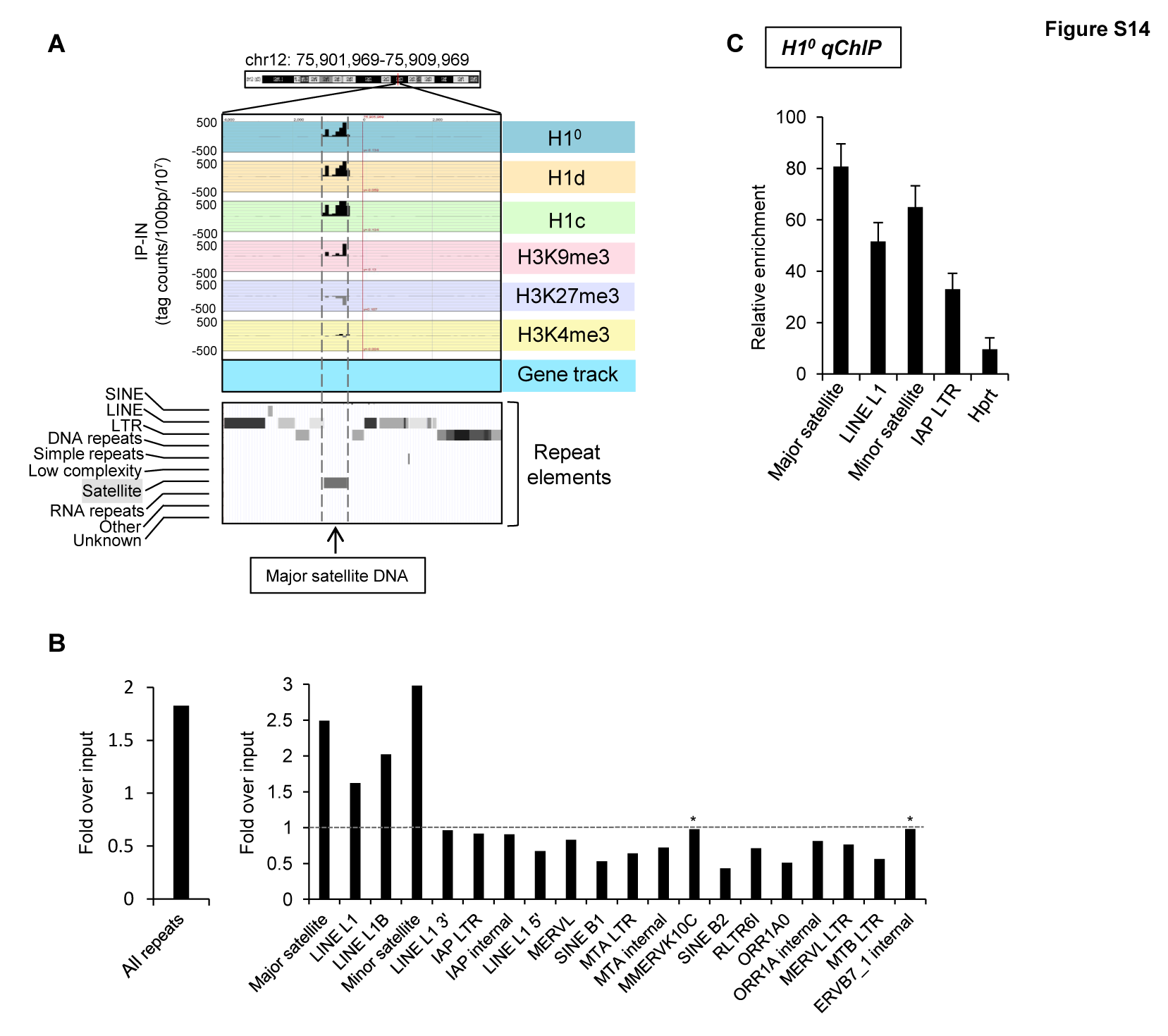

Supplement: Figure S14 — H10 is enriched at satellite sequences. (A) A typical peak region of H10 at major satellites. (B) Fold enrichment of percent mappable repeats from the H10 ChIP-seq library over that of input-seq library on total repeats (left) and 20 most abundant repetitive sequences (right). P values calculated with Fisher's exact test comparing ChIP-seq with input-seq libraries are less than 1.8×10^−21^ for all repeats shown except those marked with “*”. *: P>0.01. (C) qChIP analysis of H10 occupancy at selected repetitive sequences. Relative enrichment was calculated by normalizing the signals of ChIP over that of IgG. (TIF) [file pgen.1003417.s014.tif]

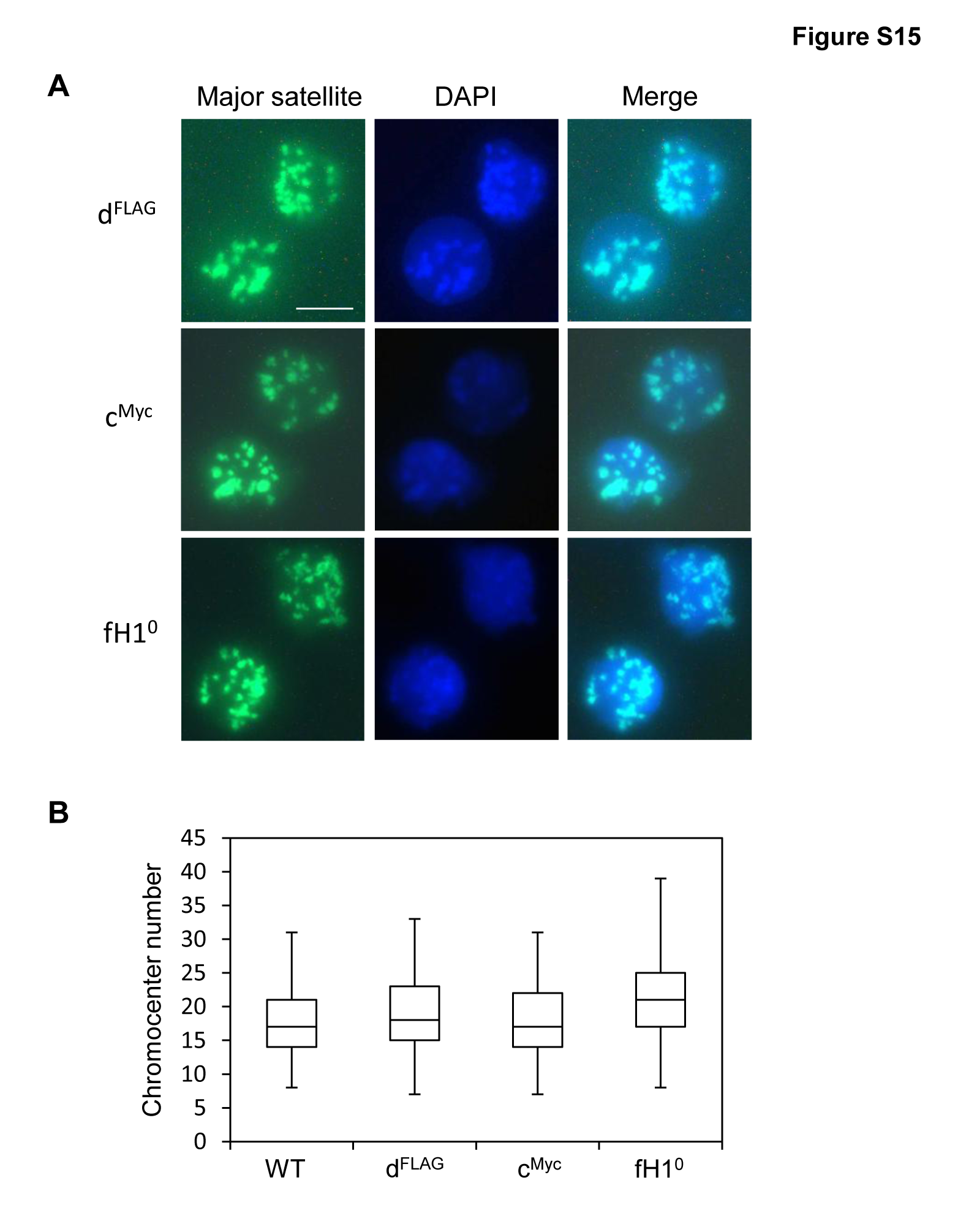

Supplement: Figure S15 — FISH analyses of chromocenters in H1dFLAG, H1cMyc, and fH10 ESCs. (A) Typical FISH images of indicated cells hybridized with a major satellite probe are shown in left panels. DNA was counterstained with DAPI (middle), and merged images are shown in right panels. Scale bar: 10 µm. (B) Box plots of the numbers of chromocenters in indicated ESCs. The line in the box indicates the median, while the bottom and top of the boxes are the 25th and 75th percentiles, respectively. (TIF) [file pgen.1003417.s015.tif]

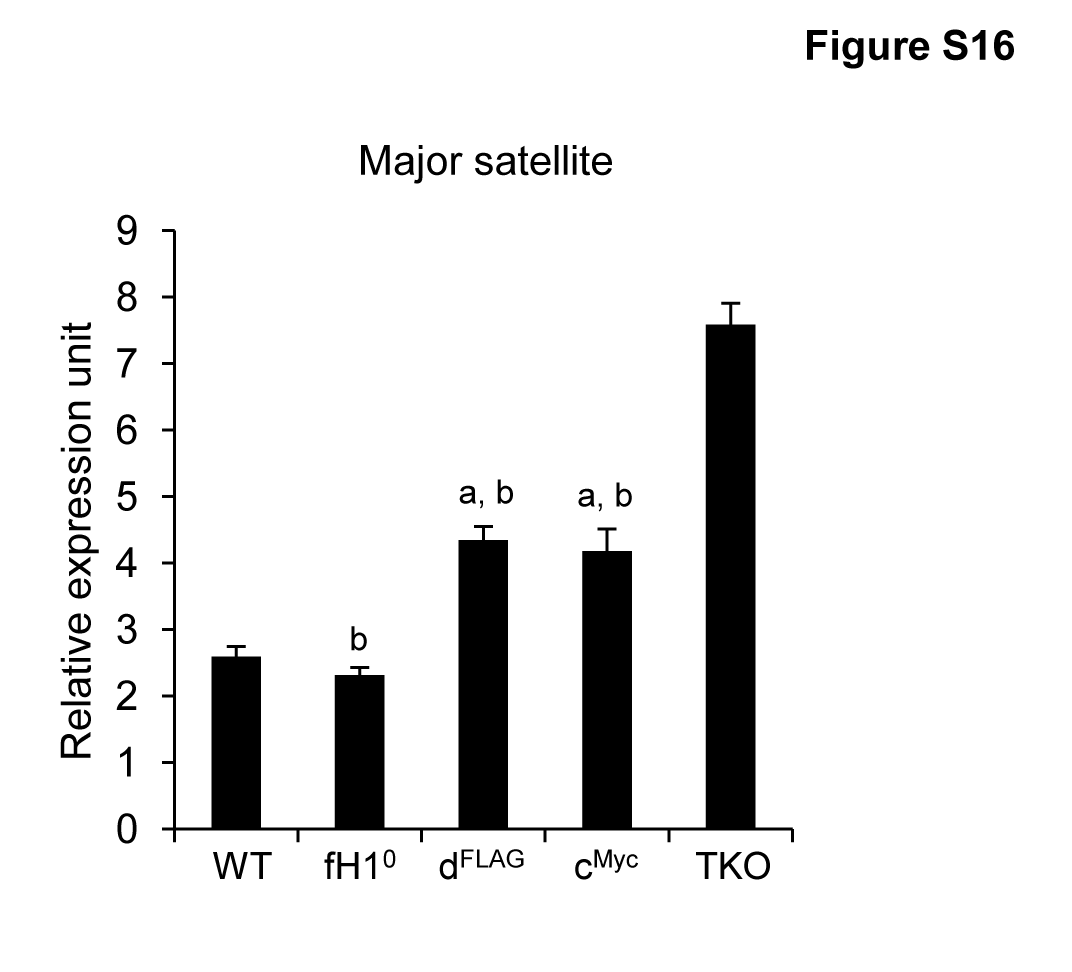

Supplement: Figure S16 — Expression analysis of major satellites in fH10, H1dFLAG, and H1cMyc ESCs. Data are shown as mean ± S.D. a: P<0.05 in comparison with WT; b: P<0.05 in comparison with TKO. (TIF) [file pgen.1003417.s016.tif]
