# Supplementary material for: High-Resolution Mapping of H1 Linker Histone Variants in Embryonic Stem Cells
Source: PLoS Genet. 2013 Apr 25;9(4):e1003417. doi: 10.1371/journal.pgen.1003417 (PMC3636266; doi:10.1371/journal.pgen.1003417)
Supplement: Table S1 — List of read length, counts, and total mappable reads (to mm9) of the libraries. (DOC) [file pgen.1003417.s017.doc]

| Library | Read length (bp) | Total reads | Mappable reads | % mappable |
| --- | --- | --- | --- | --- |
| Input-a | 85 | 33,441,224 | 29,809,507 | 89.14 |
| H3K4me3-1 | 85 | 22,142,352 | 19,972,402 | 90.2 |
| H3K4me3-2 | 104 | 29,439,221 | 24,864,366 | 84.46 |
| H3K27me3-1 | 85 | 36,662,043 | 31,760,328 | 86.63 |
| H3K27me3-2 | 104 | 30,530,476 | 27,333,935 | 89.53 |
| Input-b | 104 | 58,956,901 | 55,637,627 | 94.37 |
| H1d-1 | 104 | 128,728,185 | 113,872,952 | 88.46 |
| H1d-2 | 104 | 88,243,326 | 72,421,297 | 82.07 |
| H1d-3 | 100 | 8,982,438 | 8,257,555 | 91.93 |
| H1d-4 | 100 | 6,338,749 | 5,819,605 | 91.81 |
| H1c-1 | 104 | 78,246,028 | 70,695,286 | 90.35 |
| H1c-2 | 100 | 7,159,846 | 6,463,193 | 90.27 |
| H3K9me3-1 | 104 | 83,327,029 | 78,019,097 | 93.63 |
| H3K9me3-2 | 100 | 12,369,001 | 11,633,045 | 94.05 |
| H3K9me3-3 | 100 | 44,923,673 | 36,307,313 | 80.82 |
| H1d-*Trans*-1 | 100 | 6,889,836 | 6,279,397 | 91.14 |
| H1d-*Trans*-2 | 100 | 64,330,869 | 50,757,056 | 78.9 |
| H1^0^ | 100 | 42,232,533 | 37,215,308 | 88.12 |

**Table S1. List of read length, counts, and total mappable reads (to mm9) of the libraries.**
